# Ageing in the brain: mechanisms and rejuvenating strategies

**DOI:** 10.1007/s00018-023-04832-6

**Published:** 2023-06-24

**Authors:** Filipa Gaspar-Silva, Diogo Trigo, Joana Magalhaes

**Affiliations:** 1 i3S-Instituto de Investigação e Inovação em Saúde, Universidade do Porto, Rua Alfredo Allen 208, 4200-135 Porto, Portugal; 2 Institute of Biomedicine (iBiMED), Department of Medical Sciences, University of Aveiro, 3810-193 Aveiro, Portugal

**Keywords:** Ageing, Brain, Neurodegeneration, Cognition, Rejuvenation

## Abstract

Ageing is characterized by the progressive loss of cellular homeostasis, leading to an overall decline of the organism’s fitness. In the brain, ageing is highly associated with cognitive decline and neurodegenerative diseases. With the rise in life expectancy, characterizing the brain ageing process becomes fundamental for developing therapeutic interventions against the increased incidence of age-related neurodegenerative diseases and to aim for an increase in human life span and, more importantly, health span. In this review, we start by introducing the molecular/cellular hallmarks associated with brain ageing and their impact on brain cell populations. Subsequently, we assess emerging evidence on how systemic ageing translates into brain ageing. Finally, we revisit the mainstream and the novel rejuvenating strategies, discussing the most successful ones in delaying brain ageing and related diseases.

## Introduction

Ageing is characterized by the accumulation of molecular and cellular damage over an organismal life span, and is associated with physical deterioration and an increased risk for developing diseases, including neurodegenerative disorders [1]. The inability to repair damage leads to impaired physiological functions, ultimately leading to disease and death. Twelve hallmarks have been defined as common denominators for mammalian ageing: genomic instability, telomere attrition, epigenetic alterations, loss of proteostasis, disabled macroautophagy, deregulated nutrient sensing, mitochondrial dysfunction, cellular senescence, stem cell exhaustion, altered intercellular communication, chronic inflammation and dysbiosis [2]. Interestingly, the established hallmarks of ageing are also highly associated with an increased risk to develop neurodegenerative diseases [3].

Being composed of mitotic, but also by postmitotic cells, the brain is particularly sensitive to the effects of ageing, manifested as structural and cognitive alterations [4, 5]. With age, there is a natural progressive decline in memory and learning capability, as well as decreased decision-making speed, sensory perception, and motor coordination [6]. Whereas some individuals have a healthy brain ageing trajectory, many develop age-associated diseases; in fact, the prevalence of neurodegenerative hallmarks in the brains of older populations is extremely common, but the susceptibility to developing age-related diseases is greatly dependent on genetics and environmental factors [1].

The development of age-related neurodegenerative diseases has devastating effects to the elder population, depriving individuals from their memories, disrupting their social behaviour, and eventually taking their autonomy. Understanding the molecular mechanisms behind brain ageing will support the development of strategies to delay ageing and prevent or even treat age-related neurodegenerative diseases. In this review, we present the current knowledge in the molecular mechanisms of ageing in the brain, discuss how systemic ageing impacts the brain, and, finally, review the latest published rejuvenating strategies and their impact in brain.

## Molecular mechanisms of brain ageing

**Fig. 1:**
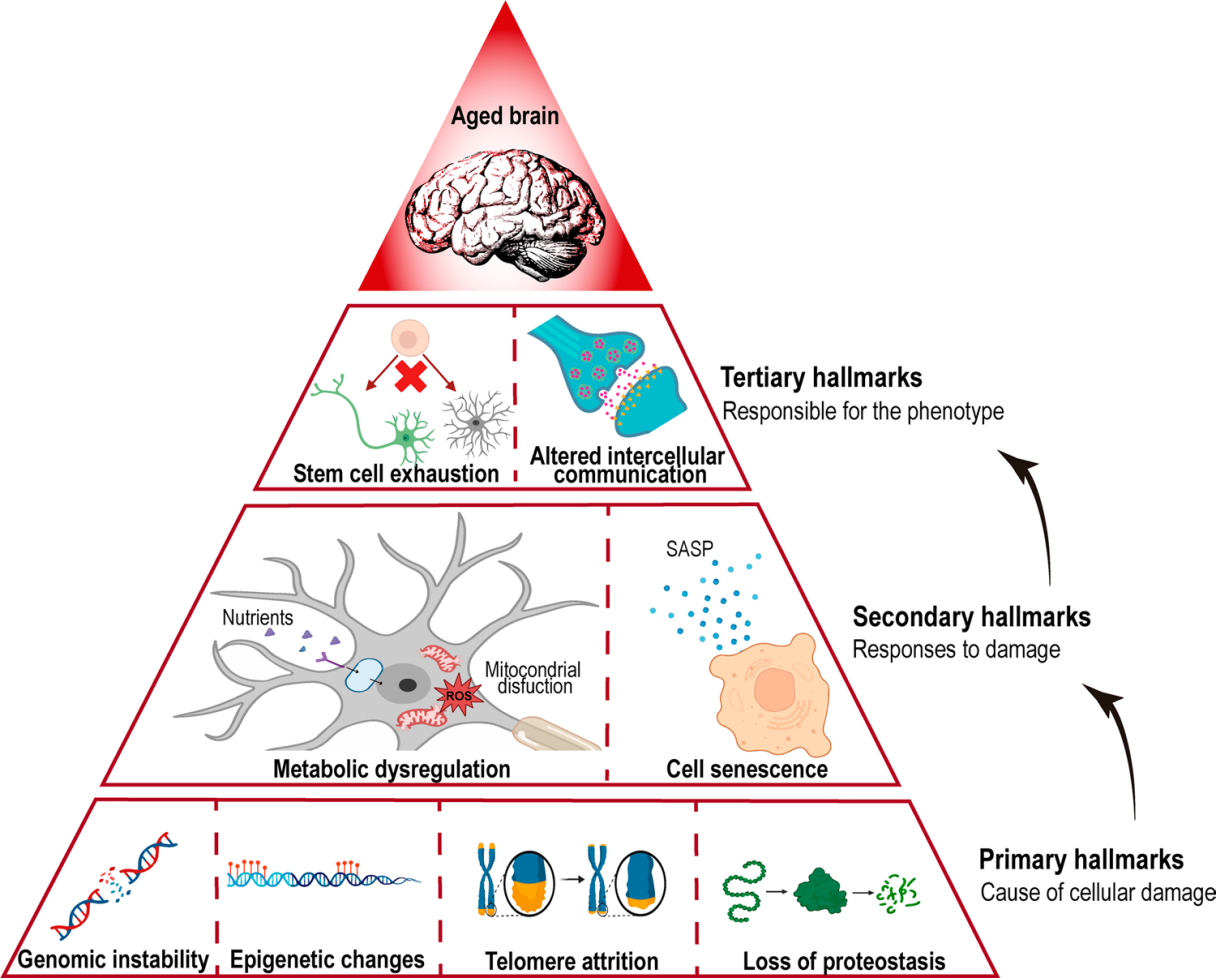
Hallmarks of brain ageing. Scheme depicting the hallmarks of ageing in the brain described in this review: genomic instability, telomere attrition, epigenetic alterations, loss of proteostasis, dysregulated metabolism (including nutrient sensing and mitochondrial dysfunction), cellular senescence, stem cell exhaustion, and altered intercellular communication. The pyramidal illustration represents the primary hallmarks (on the bottom), antagonistic (secondary) hallmarks emerging in response to the primary hallmarks, and the tertiary integrative hallmarks, ultimately leading to tissue homeostasis failures and dysfunction

At a molecular and cellular level, several pathways and biomarkers are associated with ageing; Lopez-Otin et al. described and categorized the hallmarks that reflect the mammalian ageing process [2]; the brain ageing hallmarks [6] mostly overlap with the classic ageing hallmarks [2, 7]. The primary hallmarks cause damage to cellular function, and include genomic instability, telomere attrition, epigenetic alterations, loss of proteostasis and disabled macroautophagy. These are followed by the secondary (or antagonistic) hallmarks that are responses to damage, including metabolism dysregulation: nutrient sensing, mitochondrial function impairment, and cellular senescence. Finally, the tertiary, integrative hallmarks are triggered: these are related to tissue homeostasis failures, and include stem cell exhaustion, impairment of intercellular communication and chronic inflammation, which lead to systemic decline and dysfunction (Fig. 1).

### Genomic instability, DNA damage, epigenetic changes, and telomere attrition in brain ageing

DNA damage increases with age [8]. Postmitotic neurons, with highly demanding energy requirements, elevated transcription, and long life span, are highly susceptible to DNA damage. To counteract this, neurons are equipped with efficient DNA damage response (DDR) pathways, but impairment or overwhelming these pathways, due to ageing, leads to unrepaired DNA lesion accumulation, resulting in genomic instability, transcription dysregulation, and, consequently, a cascade of events culminating in cell senescence or cell death [9]. DNA damage is highly associated with neuronal dysfunction and neurodegeneration, and is an early indicator of neuropathology [9–11]. Additionally, although post-mitotic neurons are the most susceptible, glial cells have also been shown to suffer pathological DNA damage during ageing in mice [12–14]. Recently, histone deacetylase 1 (HDAC1) was shown to be involved in oxidative DNA damage in brain ageing and Alzheimer’s disease (AD), with mice lacking HDAC1 displaying age-associated DNA damage accumulation and cognitive impairment [14].

During ageing, epigenetic modification occurs, with DNA methylation sites changing, relatively consistently between individuals and across species, allowing the identification of a series of individual methylation sites, now called DNA methylation clocks. These can be used as biomarkers of healthy ageing or disease risk, or to determine the efficiency of therapeutic interventions [15, 16]. DNA methylation is associated with transcription repression [17, 18], which, in principle, can be highly detrimental; however, the exact role of DNA methylation in brain ageing is still unknown, and it remains undetermined whether methylation alterations are a cause or a consequence of ageing. Analysis of DNA methylation patterns of different neurodegenerative disorders, such as AD, dementia with Lewy bodies, Parkinson’s disease (PD), and AD associated with Down’s syndrome, found similar DNA methylation landscapes on post-mortem human brain tissues [19]. More recently, a genome-wide DNA methylation meta-analysis also found shared associations across neurodegenerative diseases using human plasma samples [20].

Epigenetic alterations can potentiate ageing-associated abnormalities, as exemplified in the case study of monozygotic twins discordant for AD, which revealed significant differences on the methylome [21]. Considering the open question of what determines a healthy versus a pathological ageing trajectory, the epigenome could be the answer. Regardless, in the future, epigenetic clocks could be considered as early markers of ageing and able to distinguish healthy and pathological ageing.

Another well-defined hallmark of ageing is telomere attrition. Telomeres maintain genomic stability by protecting chromosomal ends from degradation, with telomerase having a crucial role in maintaining telomere length [22]. With ageing, accumulative cell divisions and exposure to stress leads to telomere shortening; when a certain critical length is reached, cell-cycle arrests and the cell becomes senescent [23]. Senescent cells, as discussed in a subsequent section, are highly detrimental [24]. In the brain, the telomere attrition hallmark concept is challenging, as evidence is currently inconsistent: literature describes telomere shortening and decreased telomerase activity in aged rat microglia, but not in astrocytes [25]; concerning neurons, recent literature shows that telomerase can persist in adult neurons and also be induced by different insults [26, 27]. As expected, high telomerase activity is found in neuronal stem cells niches in the adult mouse brain [28], being essential for cell proliferation, neuronal differentiation, neuronal survival, and neurogenesis [29]. Studies associating telomere shortening and cognitive performance, or even neurodegenerative diseases, have been conflicting [30]; nevertheless, recent in vivo studies show a promising effect of telomerase in AD [31] and PD mouse models [32, 33] and in aged mice [34]. Nonetheless, the importance of telomeres in brain ageing and the development of neurodegenerative pathways is still poorly understood and warrants further investigation.

### Loss of proteostasis

Proteostasis is the mechanism of proteome homeostasis maintenance, regulating protein turnover and structure. The accumulation and aggregation of misfolded and unfolded proteins is characteristic of ageing, as observed with the accumulation of myelin fragments in mice ageing glia [35], and is believed to contribute to progressive neuronal dysfunction and neurodegeneration [36].

Proteostasis upregulation promotes synaptic plasticity [37] and delays age-related cognitive decline in mice [38]; this process is not limited to neuronal cells, as decreasing autophagy in astrocytes and microglia compromises synaptic function and impairs mouse cognition [39].

In addition to protein deposits, other macromolecule complexes (including carbohydrates and lipids) accumulate in the ageing brain, both extracellularly (such as *corpora amylacea* [40]) and inside neurons or glial cells (such as lipofuscin [41]); the role of these aggregates is not yet clear, but are believed to affect neuronal function [42]. Several age-related neurodegenerative disorders clearly feature protein unfolding and aggregation, notably β-amyloid and Tau in AD, α-SYN in PD, superoxide dismutase in amyotrophic lateral sclerosis (ALS), huntingtin in Huntington's disease (HD), or prion protein (PrP) in prion diseases, but although the correlation between aggregation and disease is very well established, as we have recently reviewed [43], causality has not so easily been determined; in fact, some of the neurons with aggregated inclusions were found not to be the ones degenerating in HD, and vice versa [44, 45]; nonetheless, evidence suggests that improved proteostasis has beneficial effects in ageing: upregulation of proteostasis via heat-shock proteins [46] or the stress response pathway [47] increases life span in *Drosophila melanogaster* and *Caenorhabditis elegans*, respectively; and is being explored as a cognitive-restoring therapy in dementia [38, 48].

Being highly correlated with neurodegeneration, the increased production and impaired clearing of misfolded proteins results in accumulation of aggregated proteins with ageing, but it remains a current issue of debate whether aggregation is just a physiological consequence of ageing or is a direct contributor to ageing-related pathology.

### Metabolic dysregulation

Due to the brain’s elevated energetic demands, it is particularly susceptible to metabolic dysfunction impairment, and brain ageing is accompanied by decreased glucose availability and mitochondrial activity [6]. The recent bioenergetic hypothesis proposes that physiological ageing promotes metabolic alterations, which then lead to cognitive decline and neurodegeneration [49]; glucose metabolism and mitochondrial function are indeed impaired in the brain of most neurodegenerative pathologies [50, 51]. Ageing-associated cognitive decline can be countered by metabolic upregulation in myeloid cells [52], and therapeutic approaches targetting nutrient sensing are protective against cognitive decline [53, 54].

Neurons consume 70–80% of brain energy, requiring a continuous flow of blood glucose as an energy source, regulated by blood–brain barrier (BBB)-located glucose transporters (GLUT) [55]. Impairment of mitochondria activity during ageing results in hypometabolism and oxidative stress, and glucose hypometabolism compromises protein and neurotransmitter synthesis. Brain cognitive processing is optimized by its metabolic needs, linking cognition and energy. Indeed, network dysfunctions during cognitive ageing (aged neurons notoriously feature reduced dendritic trees, with alterations to dendritic spine size, shape, density, and turnover) are accompanied by metabolic dysfunction [56].

Although neurons are the most energetically demanding cells in the brain, mitochondria dysfunction also affects ageing glial cells. Astrocytes are recognized to play crucial roles in brain metabolism. Dysfunction of astrocytic mitochondria is linked to age-related neurodegeneration and to most age-related neurodegenerative diseases [57]. Astrocytic ageing features not only reactive oxygen species (ROS) accumulation, but also Ca^2+^ overload, as described in mice [58]. The neuroprotective capacity of astrocytes decreases during ageing, as antioxidative and mitophagic activity are decreased, while pro-inflammatory activity, via microglial signal amplification, is increased [58].

Mitochondrial DNA damage accumulates in aged microglia, accompanied by impaired autophagy, contributing to the accumulation of damaged mitochondria and potentiating ROS production; in a positive feedback loop, increased mitochondrial oxidative stress in turn activates the proinflammatory NF-κB in mice, promoting microglial ageing [59]; NF-κB inhibition in mouse microglia has a neuroprotective effect [60], but it has yet to be explored in the context of ageing-associated cognitive dysfunction.

Finally, oligodendrocytes are particularly metabolic demanding during myelination, but their energy needs remain high, due to myelin maintenance and metabolic support of neurons [61]. Ageing-related mitochondrial loss of function is anticipated to impair myelination, not only due to energy deficits and inefficient lipid syntyhesis, but also due to increased and cumulative ROS production, particularly damaging in terms of myelin lipidic oxidation [62].

Mitochondrial dysfunction is tightly associated with brain ageing, due to the accumulation of oxidative damage [63], and has been observed in hippocampal neurons [64] and linked with ageing and HD [65].These facets are mechanically related, as age-related loss of mitochondria function and efficiency results in increased neuronal oxidative stress [66], but, at the same time, impaired mitochondrial response to oxidative stress with ageing results in increased protein aggregation in human cells [67]. In addition to oxidative stress, mitochondria axonal transport is also impaired with ageing in mice [68]; moreover, cytoskeletal integrity, intimately linked to mitochondrial transport, is also altered with ageing, in mice [69], and in vitro studies using cultured rat sensory neurons showed age-related axonal viscousity changes affecting axon homeostasis [70]. Thus, not only do aged mitochondria affect axonal biology, but also they are themselves greatly impacted by the ageing axonal environment.

In parallel, ageing is also regulated by metabolic mechanisms. Nutrient signalling regulates ageing and age-related neurodegeneration [71] via the signalling pathways of the growth hormone (GH)/insulin-like growth factor (IGF), the mechanistic target of rapamycin (mTOR), sirtuins, and 5' AMP-activated protein kinase (AMPK) [72, 73].

In humans, signalling of the GH/IGF axis declines with ageing [74] and its inhibition appears to promote brain health in ageing, by increasing stress resistance, regulating metabolism, and reducing inflammation. IGF1 may have opposing effects in the ageing brain, depending on context: its inhibition appears to be neuroprotective against protein aggregation (via proteostasis upregulation), but IGF1 silencing is on the other hand linked to cognitive dysfunction and neurovascular disruption [75]. Balancing this dual effect is required to efficiently develop therapeutic strategies.mTOR is a serine–threonine kinase signal integrator that can influence longevity and ageing, highly expressed in neurons [76] and glia [77]. Its role in energy metabolism, autophagy, and proteostasis regulation [78] is essential for brain homeostasis during ageing [79]. Systemic mTOR activity increases during ageing, impairing autophagy [80], but is decreased in the ageing mice hippocampus [81], and research is clarifying its role in the ageing brain.

Sirtuins ameliorate autophagy dysfunction [82], neuroinflammation [83], and mitochondrial dysfunction in the ageing brain [84]. Sirtuin levels diminish with ageing [85] and, together with mTOR, are promising targets to increase life span [86]. The members of the sirtuin family most commonly linked to ageing are the mitochondrial SIRT3/4/5. Their activity depends on nicotinamide adenine dinucleotide (NAD^+^), whose levels seem to decrease with ageing, leading to mitochondrial deterioration and sirtuin activity reduction [87]. Moreover, SIRT3 was shown to be critical for neuronal proliferation in cell culture [88] and long-term memory function and cognition in the ageing mouse brain [89]

Tightly related to the previous signalling pathways, AMPK was described to slow down ageing and increase life span in *C. elegans* by modulating the mitochondrial network [90], with its signalling declining with age. AMPK upregulates brain derived neurotrophic factor (BDNF), essential for synaptic transmission and memory consolidation [91] and plays neuroprotective roles by mitigating neuroinflammation and oxidative stress, being explored as a clinical target to promote longevity [92].

Age-related metabolic and mitochondrial regulators are becoming increasingly established as key triggers for cognitive decline and are potential future therapeutic strategies [53].

### Cellular senescence in the aged brain

Originally described as an irreversible cell cycle arrest in proliferative cells, cellular senescence can be elicited by various intrinsic and extrinsic stimuli and developmental signals [93], but most importantly, their abundance is increased in ageing and progeroid syndromes [94]. Senescent cells undergo alterations in morphology, gene expression (transcriptional and epigenetic alterations), and metabolic activity; these cells are characterized by chronic inflammation, heightened oxidative stress, persistent DNA damage response, cell cycle arrest, and chromatin reorganization, developing a senescence-associated secretory phenotype (SASP) [95]. SASP is characterized by cytokine, chemokine, growth factor, and protease secretion [96], and senescent cells have a pleiotropic effect on tissue microenvironment via these remodelling factors, inducing chronic sterile inflammation, leading to detrimental phenotypes in nearby cells and tissue dysfunction, and contributing to age-related diseases [24].

Removal of senescent cells was shown to increase life span in mouse models [97, 98]. In the brain, various cell populations, including astrocytes, microglia, and oligodendrocytes present senescence [99]. Neurons are of particular interest: being non-mitotic, they exhibit a senescence-like phenotype associated with ageing and neurodegenerative phenotypes [100, 101], but since cellular senescence was first described as a permanent cell cycle arrest, the first evidence of neuronal senescence features was puzzling. Nevertheless, several independent studies have described a senescence-like behaviour and senescence biomarkers in neurons [100–102], although their molecular mechanisms of induction remain poorly understood.

It is also important to mention the increased senescence in stem cell niches during ageing, including in the brain, an important contributor for neurogenerative diseases and cognitive impairment [103].

Cellular senescence can trigger neurodegeneration via an array of mechanisms, including inflammation, through SASP, but also mitochondrial dysfunction, oxidative stress, disrupted protein homeostasis, and compromised nuclear and blood brain barrier (BBB) integrity. By persisting in the brain, senescent cells contribute to cognitive decline by impairing synaptic function, inducing paracrine inflammation and senescence. Growing evidence points to a close association between senescence and neurodegenerative diseases, such as AD and PD [100, 104–108], and eliminating senescent cells, using senolytics, not only improves brain ageing phenotypes in mice and rats [109, 110], but is also beneficial in several models of neurogenerative diseases [104, 106, 111].

### Stem cell exhaustion in brain ageing

One of the most striking characteristics of ageing is the loss of tissue regenerative capability, with evidence suggesting a correlation between stem cell dysfunction, DNA damage accumulation, telomere shortening, and senescence [7]. In the brain, adult neurogenesis is limited to the subgranular zone (SGZ) in the hippocampal dentate gyrus, and the subventricular zone (SVZ) of the lateral ventricles, and it is compromised during ageing, with decreased and more dormant neural stem cells, decreased neuronal fate commitment, and decreased self-renewal and survival [112]. Additionally, aged neural stem cells present hallmarks of ageing, including epigenetic alterations, proteostasis dysregulation, senescence, and inflammation [113].

In rodents, loss in neurogenesis is observed early in the mature brain [114, 115], but the concept of neurogenesis decline in humans is controversial [116]. While some studies describe a drop in neurogenesis in adult human brains [117], others report stable neurogenesis in older, healthy individuals [118]. More recently, neural stem cell subpopulations were shown to undergo asynchronous decline exhibiting early molecular ageing [114]. Neurogenesis impairment with age was shown to accompany a decrease in learning and memory [119], cognitive impairment, and neurodegenerative diseases, one example of which is the evidence of reduced neurogenesis in AD [120, 121]. Even though there are inconsistent reports linking neurogenesis and cognition [122], a potential role in age-related neurodegeneration can be proposed.

### Impairment of intercellular communication

Ageing features chronic low inflammation (inflammaging) due to the accumulation of tissue damage, dysfunction of the immune system, accumulation of pro-inflammatory cytokine secreting senescent cells, and defects in the autophagy system [7].

Glia, a brain proliferative cell population, is thought to be the main contributing component for inflammaging in the aged brain, as both astrocytes and microglia become more senescent with ageing, experiencing an increase in inflammatory profile and dysfunction [99]. Glial dysfunction impacts normal brain homeostasis and is associated with cognitive decline through senescence and proinflammatory secretory mediators [110]. Age-related brain inflammation induces synaptic alterations and changes in neuronal function [123], but the mechanism leading to cognitive impairment during ageing is multifactorial; nevertheless, inflammaging seems to have a significant role in age-related cognitive decline and the development of neurodegenerative diseases [123–125].

Waste clearance is essential for brain homeostasis and function. With ageing, the mechanisms responsible for clearing metabolic by-products and cellular debris are compromised; this impairment is associated with cognitive alteration and neurodegeneration [126].

The brain lacks a conventional lymphatic system, but a novel system for brain waste clearance was recently described, the glymphatic (glial-lymphatic) system, that facilitates the metabolic clearance in the central nervous system (CNS) through the flow of interstitial and cerebrospinal fluid via perivascular pathways, controlled by astrocytes, specifically the astrocytic aquaporin-4 [127]. Glymphatic clearance is reduced in aged mice [128] and humans [129], and seems to be essential to clear neurotoxic protein aggregates, with its impairment favouring neurodegeneration [127]. Besides the glymphatic system, the CNS has other mechanisms of clearance, such as cellular uptake and transport across the BBB [130]. The dysfunction of neural cells observed in ageing will surely impact their ability to manage and clear waste; additionally, BBB is also compromised in ageing [131]. Waste accumulation can potentiate the activation of immune cells in the brain, raising its pro-inflammatory status [127].

Impairment of the adaptive stress response signalling and aberrant neuronal network activity are other crucial markers of brain ageing [6]. Because cells are constantly exposed to metabolic, ionic, and oxidative stresses, cellular stress, response systems were developed to adapt to stresses, alleviate the danger, and improve cell defenses against future stress, such as calcium and ATP levels or ROS imbalances [6].

During ageing, neuronal capability to regulate calcium dynamics is compromised [132]. The major causes of neuronal calcium dysregulation during ageing are ER stress, altered mitochondria homeostasis, dysregulated calcium channels’ activity, and altered levels of calcium-binding proteins [133].

Calcium is extremely important for regulating neuronal function, plasticity, synaptic transmission and to integrate neuronal networks; moreover, calcium is crucial for ER and mitochondria homeostasis; hence, impairment of calcium dynamics is linked to neuropathology vulnerability [133] and critical for cognition [134]. Importantly, a continued increase in intracellular calcium levels can damage and kill neurons by activating calcium-dependent apoptosis [6, 133]; restoring neuronal calcium homeostasis is enough to revert cognitive alterations in aged mice [135].

The age-dependent decline in cell metabolism results in disruption of brain energy homeostasis, with faulty neuronal glucose metabolism and ATP deficits [6]. The general energy deficiency leads to impaired energy-requiring mechanisms, such as calcium pump activity, transcription, and protein production. Additionally, ATP and calcium pumps are extremely important pieces in neuronal network activity, being crucial for action potential conduction and synaptic activity, and, ultimately, cognitive function [136].

The increase in ROS production due to mitochondria dysfunction, discussed in a previous section, leads to changes in the redox state of the cell, including the oxidative state of DNA, altering transcription factor responsiveness and even protein production and function [6]. This is intricately linked to age-associated cognitive decline, with antioxidants having a neuroprotective effect [137].

Another consequence of ageing is the loss of white matter, with evidence of decreased myelination with age caused by oligodendrocyte oxidative DNA damage [138]. Since communication between different brain regions occurs preferentially via myelinated axonal projections, myelination defects are surely detrimental for the network, leading to increased risk of neurological diseases [138, 139].

Evidence shows that brain structure integrity (with appropriate types of neurons, functional glia, and reliable neurotransmitter systems) is essential for the correct brain circuit function; ageing disturbs both cellular local mechanisms and circuit networks within the brain, leading to cognitive dysfunction.

## Differential neural vulnerability to ageing

Ageing is the leading risk factor for neurodegenerative diseases, but the precise boundaries between healthy and pathological brain ageing are not well defined. Genetic, epigenetic, and environmental factors are contributors to human ageing, and humans age at different rates [140]. In fact, recent evidence indicates that both mouse and human organs also age at different rates [141, 142], which unlocks the question why some organs and systems are more vulnerable than others. In the case of the brain, it is well known that neurodegenerative diseases, such as AD, PD, and ALS, present region-specific neurodegeneration; however, it is still unknown why some brain regions are more vulnerable to neurodegeneration [143]. In the case of physiologic ageing, evidence shows increased vulnerability of white matter (primarily myelinated axons) to age in humans [144], certainly leading to functional consequences [139]. Interestingly, the prefrontal cortex and the hippocampus display a significant decrease in volume with age in humans [145], indicating a possible higher susceptibility to ageing.

The alteration of cerebral blood flow during healthy ageing is a concept still needing further studies, but there are some indicators that cerebral blood flow is altered in ageing, exposing different brain regions to hypoxia, possibly increasing their risk of neurodegeneration [143].

Looking in more detail to the brain, each region comprises various types of neural cells, differing from region to region. This regional heterogeneity may underlie the different regional susceptibility to age-related alterations. The hippocampus has an interesting characteristic: it presents neural stem cells in the subgranular zone of the dentate gyrus, which declines in ageing [117], with functional implications in learning and memory in rodents [114]. This unique characteristic can render the hippocampus a higher susceptibility to age-related decline.

Age-related neuronal loss has been shown in the hippocampus and the substantia nigra [143, 146], supporting the notion of increased hippocampal and substantia nigral susceptibility to age-related dysfunction; interestingly, large sized neurons with lengthy axons tend to be more vulnerable to ageing [147], which agrees with neuronal features of the hippocampus and substantia nigra [143].

An alternative theory concerning neuronal vulnerability proposes that age-related mitochondrial dysfunction and oxidative stress surge are the main culprits behind the varying neuronal sensitivity, with some neurons, such as hippocampal and substantia nigral ones, being more sensitive to this damage [148]. Besides neurons, glia was shown to present differential age-associated regional alterations [149]. Consistently, hippocampal and substantia nigral astrocytes seem more susceptible to ageing, in terms of number, proinflammatory reactivity, and protective capacity, whereas microglia seem to present more age-related alterations in the hippocampus [143].

Additionally, in humans, the size and thickness of the prefrontal cortex was also shown to decrease with age [145]. The prefrontal cortex plays an important role in cognition, including attention and memory, and is interconnected with subcortical regions, such as thalamus, amygdala, and hippocampus, exerting control over other brain regions involved in cognitive function [150]. Neuronal number in some areas of the prefrontal cortex of non-primate humans was found reduced and associated with memory impairment [151]. However, more recent literature suggests that cognition alteration due to changes in the prefrontal cortex are more likely due to changes in neuronal density and synaptic plasticity and function than to neuronal loss [152]. Concerning glial cells, both microglia and astrocytes are activated in the aged prefrontal cortex, correlating with synaptic dysfunction [153, 154].

The use of single cell transcriptomic profiling technology to depict changes in brain ageing is an emerging trend that could greatly improve the current understanding of brain complexity and specific cell type and regional vulnerability observed in ageing and age-related pathology. Comparing young and old brains produced comprehensive datasets of ageing-related genes, pathways, and interactions of different neural cell types. In one such recent study using mouse brain, authors concluded that ageing, rather than inducing a universal program, leads to distinct transcriptional courses in different cell populations [155]. More recently, using spatially resolved single-cell transcriptomics, a new high-resolution cell atlas of brain ageing depicted the changes in gene expression and spatial organization of major cell types in the frontal cortex and striatum over the mouse life span [156], while other reports on single-cell transcriptomics highlight transcriptional alterations in different cell types during ageing [157, 158]. Moreover, high-resolution ageing clocks from single-cell transcriptomic data were very recently proposed [159] and will allow the quantification of transcriptomic rejuvenation of therapeutic interventions. In coming years, research using these newly developed technologies will surely help disclose the complex question of why brain regions have differential vulnerability to ageing, opening new venues for rejuvenating therapies.

## Impact of systemic ageing in brain health

**Fig. 2:**
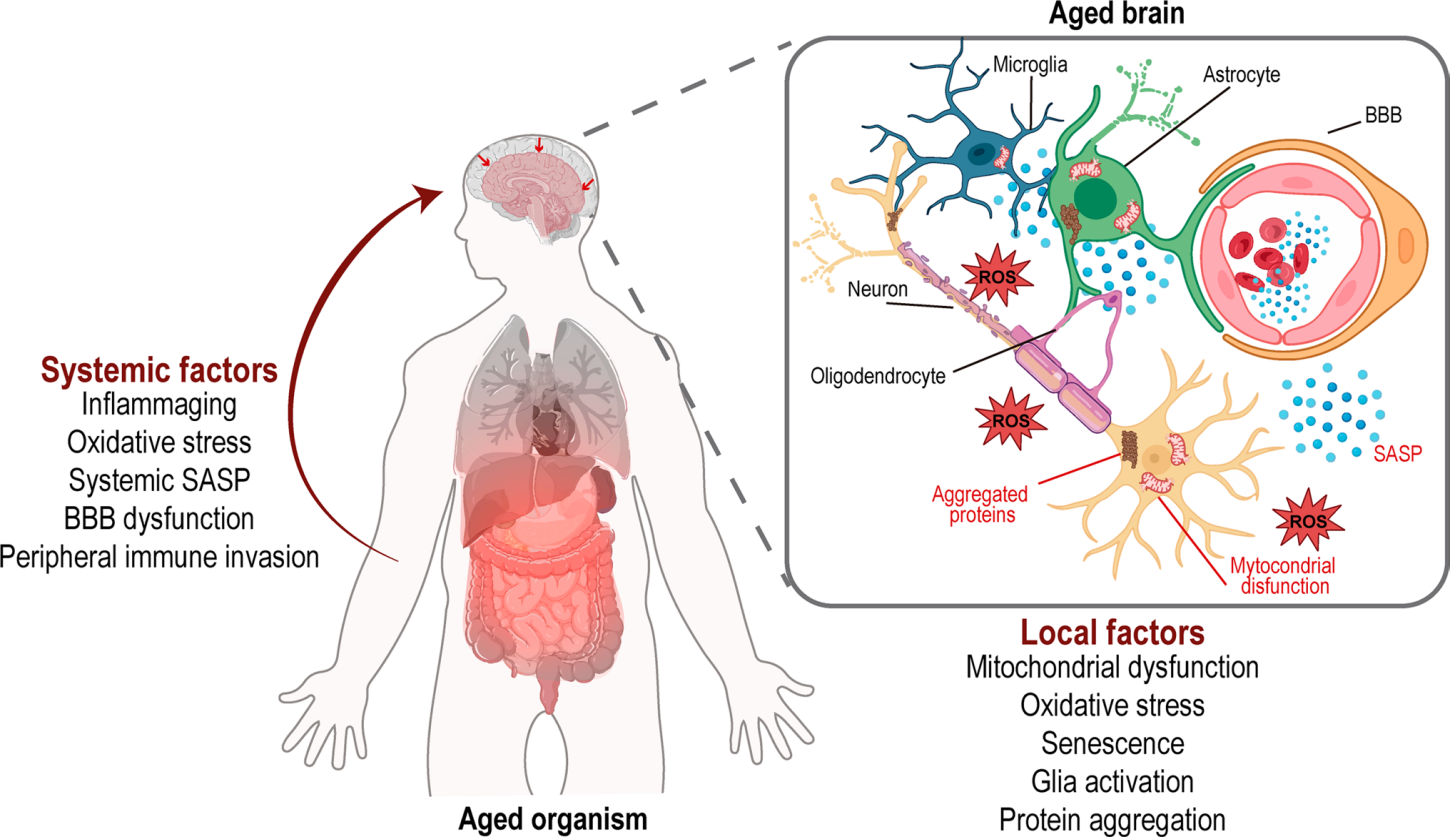
Impact of local and systemic ageing in brain health. Both neural cellular intrinsic factors and extrinsic systemic defects can contribute to the decline in brain function during ageing. Locally, brain cells can accumulate age-related damage, due to mitochondria dysfunction, oxidative stress, inflammation, or protein aggregation, which alters both neural cell survival but also core neural circuitry, and ultimately brain function. Systemic tissue ageing impacts brain health decline with age: periphery-derived factors such as inflammation mediators, SASP, peripheral immune invasion, and oxidative stress mediators that can reach the brain. *ROS* reactive oxygen species, *SASP* senescence-associated secretory phenotype, *BBB* blood–brain barrier

Besides cell-intrinsic defects in neurons and glia, leading to brain dysfunction due to age-related damage accumulation, systemic factors may also contribute to brain dysfunction and decline with age (Fig. 2). Since tissues and organs age at different rates [141, 142], it is possible that some tissues influence others’ ageing decline. Several pivotal studies reveal that young blood or bone marrow transplantation reverse age-related cognitive dysfunction and recover synaptic plasticity [160–162]. Additionally, whole body clearance of senescent cells was enough to ameliorate age-related brain inflammation and cognitive impairment in mice; however, this study targeted whole-body, and it is thus unknown whether the cognitive improvement is a result of senescence clearance in the CNS or in peripheral organs [163]. Other systemic manipulations, such as exercise [164] and caloric restriction [165], show promising results. These systemic rejuvenating approaches, especially the ones using young blood or bone marrow transplantation, are strongly consistent with the idea that systemic ageing can induce brain dysfunction.

Experimentally, the mechanistic study of how peripheral ageing leads to brain ageing is complex, as separating the periphery from the brain, in respect to mechanisms or interventions, is difficult. Nonetheless, some peripheral mediators of ageing are known to impact the brain, in particular pro-inflammatory mediators. Age-related systemic inflammation (inflammaging) is well documented to impact brain health and cognition in mice and humans [166–168]. The role of the gut in inflammaging is now well recognized; having the ability to secrete inflammatory products, the gut can affect other systems and organs, accelerating their ageing-associated decline [169]. The gut and the brain establish a bidirectional connection: the gut–brain axis, established through the vagus nerve, the immune system, and bacterial metabolites and products. Research indicates that inflammatory gut metabolites impact brain cognition and psychiatric symptoms, also seemingly accelerating neurodegenerative diseases [170, 171]. Nevertheless, the mechanisms behind the impact of gut microbiota in brain diseases are still poorly understood and need further investigations.

**Fig. 3:**
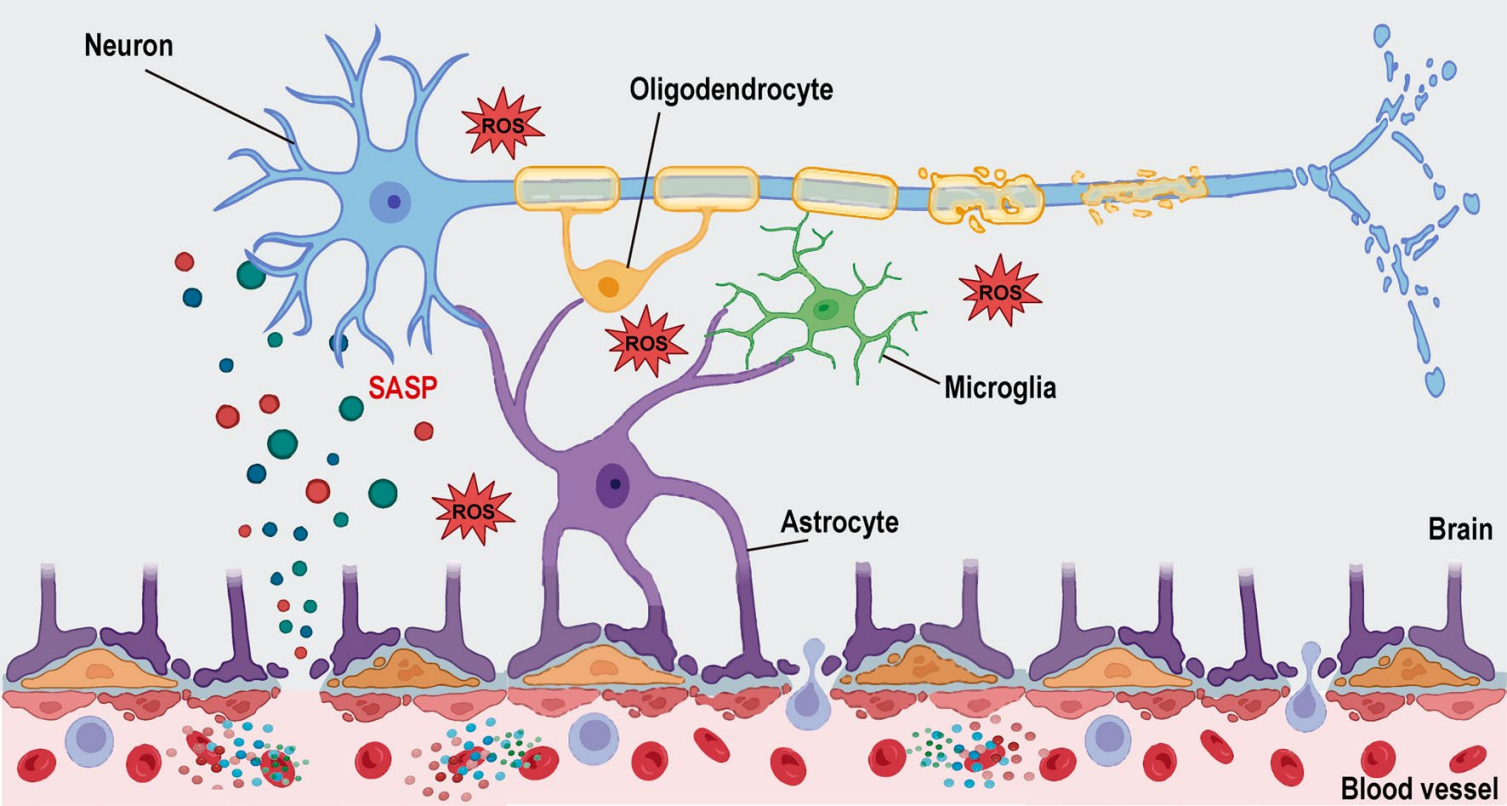
Blood–brain barrier (BBB) dysfunction in ageing. The brain is protected from the periphery by the BBB, which maintains brain homeostasis by controlling the selective crossing of molecules. The BBB involves brain endothelial cells, mural cells (pericytes and vascular smooth muscle cells), astrocytes, neurons, microglia, and the basal lamina. BBB disruption in ageing is well documented, being a probable route for systemic factors, such as inflammatory mediators, cytokines, immune cells, and other molecules, to reach the brain and induce cellular and tissue dysfunction. ROS: reactive oxygen species; SASP: senescence associated secretory phenotype

The brain is protected from the periphery by the BBB, a selective, semipermeable interface between the blood and the brain, with a crucial role in maintaining brain homeostasis by controlling the selective crossing of needed molecules and exclusion of toxins and pathogens. Correct BBB function requires a proper association between brain endothelial cells, mural cells (pericytes and vascular smooth muscle cells), astrocytes, neurons, microglia, and the basal lamina [172]. During ageing, this barrier is disrupted (Fig. 3), leaving the brain less protected, with evidence suggesting that normal breakdown (due to healthy ageing) is only detrimental following an exposure to secondary stress, such as inflammation, following which cognitive decline emerges [173]. It remains unclear how gut metabolites, inflammatory mediators, and other factors access the brain, but the BBB disruption caused by these inflammatory mediators is a possibility, alongside ageing-related BBB dysfunction and breakdown due to cell dysfunction. Pericytes, BBB transporters capable of sensing peripheral inflammation, may play a particularly central role, as pericyte loss leads to immunogenic protein leakage into the brain, and intact pericytes can also transduce inflammatory cues from the systemic environment [174].

Another mechanism for metabolites and other factors to reach the brain is through the blood–cerebral spinal fluid (CSF) barrier. In the choroid plexus, the close proximity of blood to the CSF can possibly lead to choroid secretory function and CSF composition modulation, with the blood–CSF barrier being a crucial transducer of systemic factors for the modulation of brain ageing. Interestingly, infusion of young CSF into aged brains restores oligodendrogenesis and improves memory in aged mice [175]. Direct peripheral immune cell infiltration into the brain via the choroid plexus has also been hypothesized [176], but further investigation is needed in the specific case of heathy ageing.

One interesting question that remains unsolved is why different tissues have different ageing rates. In the case of the brain, protective mechanisms, such as the BBB, immunosurveillance, and waste clearance, give this tissue a layer of protection lacking in other tissues such as skin, which is indeed one of the first aged tissues.

Considering the known effects of paracrine signalling, it is important to look at ageing and age interventions from a systemic point of view. Maintaining brain function and cognition is critical to the lifestyle of the aged individuals, and it is thus essential that anti-ageing therapy studies not only consider life span and effects on peripheral tissues, but also examine the potential positive effects in cognitive preservation.

## Impact of rejuvenating strategies in brain function

Even though ageing and death are inevitabilities, strategies to delay and potentially reverse ageing must be developed, with the aim to increase life span, but more importantly, to improve health span, which include the maintenance of cognitive health in the elderly and in the end of life. Systemic rejuvenating strategies that impact several organs and tissues, including the brain, will be pivotal.

When considering rejuvenating strategies, sex differences must be considered, as they can affect the rate of brain ageing. Literature shows accelerated epigenetic ageing in the brain tissue of men, compared to women [177] and a study using PET scans showed the female brain to be systematically neotenous, in comparison to male brain [178]. Other findings indicate a more prominent alteration of gene expression profile towards ageing in males, with downregulation of genes related to protein processing and energy production [179]. This highlights the importance of sex influence in ageing and shows the importance of conducting studies in both men and women disclosing the sex influence of any therapeutic approach.

**Fig. 4:**
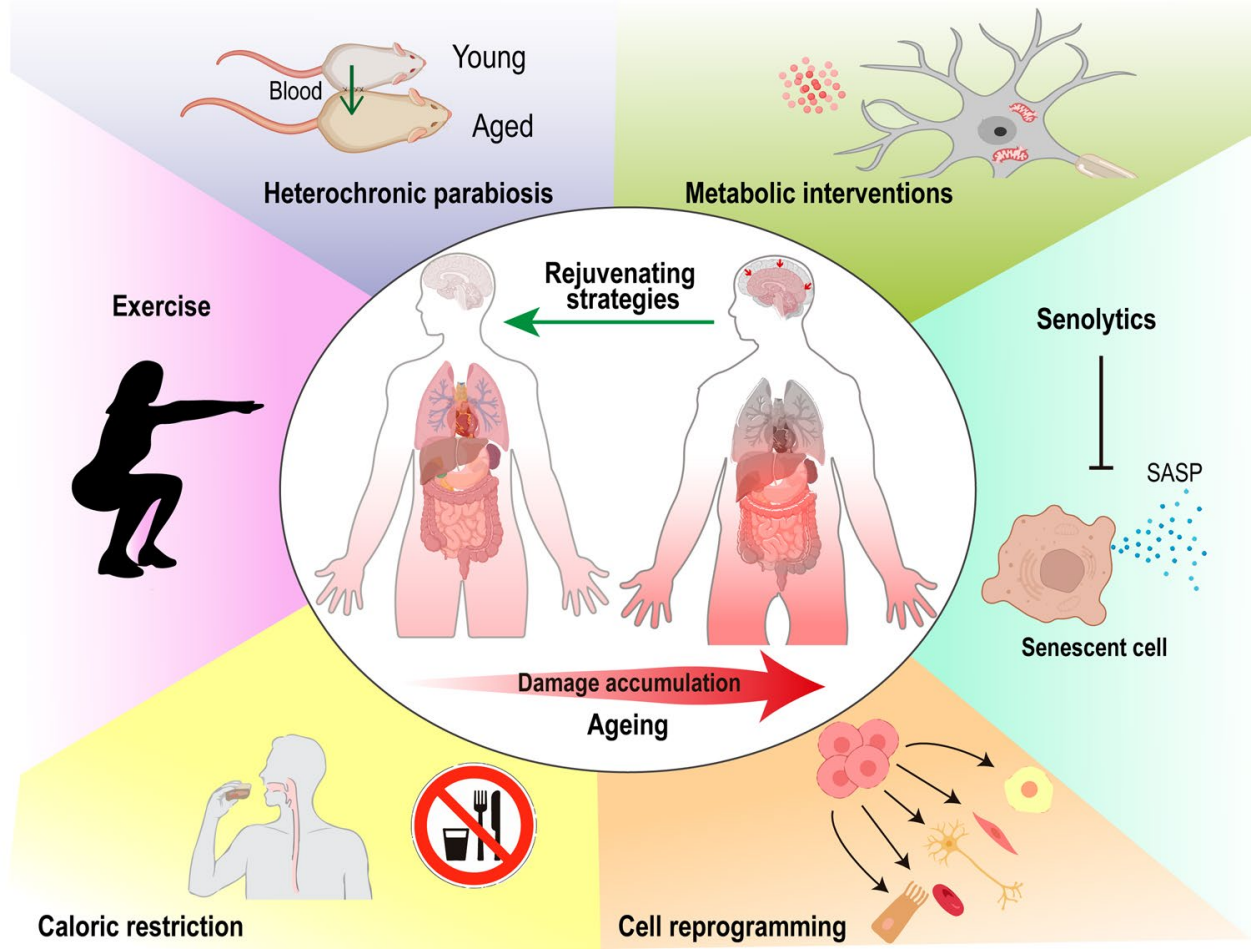
Rejuvenating strategies to improve brain health. Scheme illustrating systemic strategies found to be beneficial for brain health in ageing. With ageing comes damage accumulation in the organism, leading to increased organismal tissue dysfunction and inflammation. The brain is affected by the age-related accumulation of peripheral damage and also local damage, leading to morphological and functional alterations. Therapeutic interventions, such as heterochronic parabiosis, exercise, caloric restriction, counteracting senescence, cellular reprogramming and metabolic interventions, have a positive effect counteracting the damage accumulation that leads to brain ageing by acting systemic and/or locally in the brain

In this review, we conduct a global portrait of systemic rejuvenating strategies with a known positive impact in brain ageing and cognition (Fig. 4).

### Heterochronic parabiosis

Heterochronic parabiosis provides evidence that blood factors influence organismal ageing. In heterochronic parabiosis, the circulating systems of young and aged mouse are fused, aiming to manipulate the plasma proteome of the older animals. Exposure to old blood via heterochronic parabiosis or by administrating old blood plasma decreases hippocampal neurogenesis, decreases synaptic plasticity, promotes microgliosis, and impairs learning and memory [131], whereas exposing aged mice to young blood enhances hippocampal neurogenesis and increases dendritic spine density, improving learning and memory [160]. These results suggested the presence of “pro-cognitive” factors in young blood, and GDF11 was indeed found to mimic the effects of young blood in enhancing neurogenesis [161]. Another factor, the tissue inhibitor of metalloproteinases 2 (TIMP2), a blood-borne factor enriched in human umbilical cord plasma, was shown to improve synaptic plasticity and hippocampal-dependent cognition in aged mice [180].

The transplantation of young bone marrow was shown to rejuvenate the hematopoietic system, preserving cognitive function in old mice [162], while exposure to an old hematopoietic system was enough to elicit a decrease in hippocampal synaptic density and age-related cognitive impairment [181], defining the importance of the hematopoietic system in rejuvenation strategies for brain function. A different work showed osteocalcin to be necessary for the beneficial effect of young plasma in memory and anxiety-like behaviours in old mice, identifying it as another “pro-cognitive” young plasma factor [182]; thrombospondin-4 and SPARCL1, present in blood, were also found to mediate the beneficial effects of young blood in in vitro synapse formation [183]. Additionally, both C–C motif chemokine ligand 11 (CCL11) and β2-microglobulin have been described as pro-ageing blood factors [162].

While the administration of old blood leads to impaired cognition in mice [131], the question of how this occurs remains unsolved; a recent study implicated the vascular cell adhesion molecule 1 (VCAM1): systemic administration of anti-VCAM1 antibody or genetic ablation of Vcam1 in brain endothelial cells of the BBB was able to counteract the effects of old plasma, reverting microglial reactivity and cognitive deficits [184].

Research in parabiosis, young plasma administration, and pro-ageing/pro-youthful blood factors has demonstrated the potential that changing old blood composition in the periphery can have on cognitive function. This represents a potential for therapeutic avenues aimed at restoring CNS functions by promoting a more youthful systemic environment.

### Exercise

Exercise is a well-established modulator of beneficial effects in brain, improving neurogenesis, brain plasticity, and cognitive function, being protective for neurodegeneration [185, 186]. The positive effects of exercise are, in part, mediated by changes in the systemic environment. The skeletal muscle undergoes dramatic changes due to exercise and secretes a wide variety of molecules, including myokines that can affect other organs [187]. Lactate, a metabolite that accumulates with exercise, promotes brain angiogenesis, a possible contributor to cognitive maintenance during normal ageing [188]. Additionally, elevated levels of circulating vascular endothelial growth factor and IGF1 due to exercise were shown to mediate neurogenesis in mice [189].

Liver metabolism also endures brain-modulating alterations during prolonged exercise: under glucose depletion, ketones are produced, including the BBB-crossing β-hydroxybutyrate, which was shown to induce BDNF expression in the mouse hippocampus [190]. BDNF is a trophic factor associated with synaptic plasticity, cognitive improvement, and the alleviation of depression and anxiety [91]. Recently, other liver-derived soluble factors released during exercise were found to lead to neurogenesis and cognitive improvements on the aged brain [191], and several protein and peptide myokines were shown to regulate brain function, such as cathepsin B, clusterin, and Irisin [187].

Exercise also acts directly in the brain, namely by enhancing neurogenesis [185], enhancing autophagy [192], promoting proteostasis [193], increasing neurotransmitter levels [194], and maintaining the BBB, the neurovascular unit, and promoting glymphatic clearance [195]. It can also be beneficial for cognition by altering epigenetic markers, such as DNA methylation, histone modifications, and microRNAs (miRNAs), thus having a positive effect in brain ageing and age-related neurodegenerative diseases [196].

Exercise has the potential to modulate the local brain environment directly and via systemic targeting and is a plausible effective strategy to reverse the functional impairments of ageing in the CNS.

### Caloric restriction

A reduction of 20–40% of caloric intake was shown to counteract age-associated phenotypes, increasing life span and health span across a spectrum of species [197], including humans [198].

How dietary caloric restriction increases life span and health span is not yet fully characterized; however, metabolic increase, reduction of oxidative stress, and increased ability to counteract DNA damage, as well as beneficial immune and neuroendocrine effects, may be responsible [199]. In fact, caloric restriction is generally accepted to alter the activity of common key metabolic pathways, namely mTOR, IGF, and sirtuins [72], and to decrease oxidative damage to cellular macromolecules (including mitochondrial DNA) [200]. Moreover, age-related DNA oxidative damage is significantly reduced by caloric restriction, that acts on DNA repair by enhancing pathways such as NER, BER, and double-strand break repair [201]. A role for caloric restriction in immune regulation and ameliorating immunosenescence has been shown in mice [202] and in humans [203], with beneficial effects in stem cell function [204, 205].

While caloric restriction can enhance all of these pathways, it is evident that it also enhances neural plasticity and cognition, reducing vulnerability to age-related neuro-dysfunction and disease [206]. Caloric restriction was shown to mitigate age-related loss of neurogenesis in the brain, preventing senescence increase normally observed in the subventricular zone of the aged mouse brain, while simultaneously diminishing age-related microglia activation and pro-inflammatory cytokine increase [207] and promoting senescent astrocyte rejuvenation [165]. This dietary intervention is also beneficial for oxidative stress: it attenuates the age-related increase in neuronal plasmalemma lipid peroxidation, protein carbonyls, and nitrotyrosine [208], and reduces oxidative modifications in mitochondrial DNA, extremely beneficial for maintaining mitochondria function [200]. Overall, the decrease in oxidative stress is clearly neuroprotective [137]; additionally, caloric restriction positively alters the levels of neurotrophic factors, such as BDNF and glial cell line-derived neurotrophic factor (GDNF), and increases the expression of IGF1 [209]; these factors together surely have a positive effect in neuronal health and even neurogenesis.

Recently, as epigenetics have been granted more attention, the effect of caloric restriction in epigenetics was explored. Studies found that caloric restriction can positively alter epigenetic marks in the brain associated with ageing, protecting neuronal health [209, 210].

Caloric restriction, besides being easy to implement, modulates a pleiotropic of pathways, making it a particularly interesting anti-brain ageing therapy.

### Counteracting senescence

The negative effects of cellular senescence are both local and systemic, due to its paracrine effect via SASP. Senotherapeutics targeting senescent cells have emerged as a strategy to fight ageing and age-related diseases. Senotherapies include senolytics, which selectively kill senescent cells, senomorphics, which promote senescent cells to behave as young cells or delay senescence progression, and immune-system mediators, that stimulate senescent cells clearance [211]. In the CNS, senolytic approaches have been shown to decrease the senescence burden and improve cognitive performance and health span in ageing and neurodegenerative mouse models [99, 212]. Elimination of senescent cells, using an inducible p16-3MR transgenic mouse model which abrogated senescence cells, was shown to improve age-related PD phenotypes, including motor deficits, selective dopaminergic midbrain neuron loss, and reduced neurogenesis [106]. More recently, genetic and pharmacologic elimination of whole-body senescent cells were shown to decrease brain inflammation and cognitive impairment in aged mice [163]. Interestingly, genetically eliminating systemic p16-senescent cells partially restores immune cell activation and infiltration to youth levels, correlated with cognitive preservation [213]. Additionally, heterochronic parabiosis decreases the burden of senescence in several tissues, including the brain [214], while plasma dilution can similarly attenuate brain senescence and inflammation, improving cognition [215].

Nonetheless, it is important to mention the physiological role of senescence in tissue regeneration, homeostasis [216], and in mammalian embryonic development [217]. In this regard, a senostatic approach using senomorphics [218] to prevent senescence accrual should be considered for studies in brain ageing. One such approach is the overexpression of forkhead box M1 transcription factor, that has a senomorphic effect and increases health span in mice [219], however little is known about its effects in the brain.

### Cellular reprogramming

Using transcription factors, it is currently possible to convert somatic cells into pluripotent stem cells [220, 221]. With reprogramming, a global remodelling of the epigenetic landscape of the cells is possible, and rejuvenation a possibility. Considering reprogramming as an anti-ageing strategy, somatic cells could be turned into pluripotent stem cells, which could then be modified or corrected before redifferentiation, generating rejuvenated cells. Two opportunities arise from cell reprogramming: in vitro rejuvenated cells or tissues to replace damaged tissues and organs, and direct reprogramming of cells in the affected tissue or organ. By restoring juvenile features to cells, it is easy to understand reprogramming as an attractive rejuvenating strategy; cell reprogramming was, in fact, shown to rejuvenate senescent and centenarian human cells and reduce the deleterious effects of ageing [222], but limitations were found when proceeding to in vivo studies, concerning teratoma formation, and limited life span has consequently been reported in mice [223]. More recently, a new protocol of single short reprogramming reportedly overcomes previous problems, successfully increasing life span in mice [224]. With this short reprogramming, Alle et al. prevented age-related tissue deterioration in skin, kidney, lung, and spleen; it would be interestingly to see if this procedure also ameliorates age-related brain dysfunction.

Concerning the benefits of cellular reprogramming in age-related cognitive decline, in vivo local reprogramming was found to ameliorate ageing features in the hippocampal dentate gyrus, improving neuronal plasticity and memory in mice [225]. Since the nervous system has limited self-repair capacity, neuronal replacement using cell reprogramming is an emerging, although technically very challenging, field. Three approaches have arisen: recruitment of neural stem cell (NSC) niches to produce neurons, reprogramming of local glial cells into neurons, and transplantation of foetal progenitor cells. Various degrees of success have emerged from studies of cellular reprogramming in animal models of neurodegenerative diseases, recently extensively reviewed elsewhere [226]. Research in this field is emerging, and potentially contributing with new ways to restore CNS function and increase health span.

### Metabolic interventions

Nicotinamide adenine dinucleotide (NAD^+^) is not only a coenzyme central to cellular energy metabolism, but also an essential cofactor for NAD^+^-dependent enzymes, such as sirtuins, CD38 and poly (ADP-ribose) polymerase, influencing many key cellular functions, including metabolic pathways, DNA repair, cellular senescence, and immune function. NAD^+^ declines with ageing [227], and its precursor, nicotinamide ribose (NR), was shown to ameliorate age- and AD-related cognitive impairment in rodents [228]. Importantly, NR supplementation in humans augmented neuronal NAD^+^ levels and modified biomarkers related to neurodegenerative pathology [229].

Resveratrol is a polyphenol that activates Sirt1 and AMPK, critical regulators of energy metabolism, and its administration reversed age-related cognitive defects in rats [230]. Notably, clinical evidence indicates that resveratrol improves cerebral blood flow, cognition, perceived performance, and Aβ40 levels in plasma and cerebrospinal fluid levels, in humans [231].

Mice fed rapamycin, which targets mTOR, were described to exhibit increased life span [232], and rapamycin is reported to ameliorate age-dependent cognitive deficits in mice [233]; however, the effect of rapamycin in human age-related cognitive impairment is yet to be established.

Years ago, metformin was shown to extend life span in mice [234], and was more recently shown to reduce the risk of cognitive impairment in patients with type 2 diabetes [235]. In aged mice, metformin is anti-inflammatory, activates AMPK, inhibits mTOR signalling, and enhances autophagy in the hippocampus, leading to better cognitive and memory function [236].

The benefits of metabolic interventions in human cognitive preservation need further clinical trials to evaluate dose, treatment duration, and effect, particularly in aged non-diabetic or non-obese cohorts.

## Conclusions

Research in brain ageing still has many unanswered questions, such as: why are some brain regions more affected than others?; what determines a healthy versus a pathological ageing trajectory?; why is the rate of ageing in the brain different from peripheral tissues?; can peripheral tissue, in fact, determine brain ageing?

In this review, we described the latest research addressing some of these questions. Brain ageing, just like organismal ageing, is a complex network of processes, with the multifaceted brain ageing mechanisms involving local age-related alterations and systemic derived factors. Locally, brain cells, including neurons, glia, endothelial cells, and stem cell niches, are exposed to age-related increase in DNA damage, proteostasis dysfunction, mitochondria damage, accumulation of ROS, and senescence; together, these factors are highly detrimental to tissue function. Additionally, it is now well recognized that factors extrinsic to the CNS can have a profound effect in brain health. As such, extrinsic, less invasive, systemic manipulations altering the systemic environment, leading to a more youthful state of blood or peripheric tissues, can effectively alter brain function and ameliorate age-related brain dysfunction, promoting brain rejuvenation.

Since life span extension is only pertinent if accompanied by health span extension, and, more importantly, by preserving brain health and cognition, finding systemic rejuvenating strategies that act simultaneously in peripheral tissues and in brain function is a valid strategy, and a major accomplishment, if achieved.

## Data Availability

Not applicable.

## References

[1] Wyss-Coray T (2016). Ageing, neurodegeneration and brain rejuvenation. Nature.

[2] Lopez-Otin C, Blasco MA, Partridge L, Serrano M, Kroemer G (2022). Hallmarks of aging: an expanding universe. Cell.

[3] Hou Y, Dan X, Babbar M, Wei Y, Hasselbalch SG, Croteau DL, Bohr VA (2019). Ageing as a risk factor for neurodegenerative disease. Nat Rev Neurol.

[4] Grajauskas LA, Siu W, Medvedev G, Guo H, D'Arcy RCN, Song X (2019). MRI-based evaluation of structural degeneration in the ageing brain: pathophysiology and assessment. Ageing Res Rev.

[5] Fjell AM, Walhovd KB (2010). Structural brain changes in aging: courses, causes and cognitive consequences. Rev Neurosci.

[6] Mattson MP, Arumugam TV (2018). Hallmarks of brain aging: adaptive and pathological modification by metabolic states. Cell Metab.

[7] Lopez-Otin C, Blasco MA, Partridge L, Serrano M, Kroemer G (2013). The hallmarks of aging. Cell.

[8] Soto-Palma C, Niedernhofer LJ, Faulk CD, Dong X (2022). Epigenetics, DNA damage, and aging. J Clin Invest.

[9] Welch G, Tsai LH (2022). Mechanisms of DNA damage-mediated neurotoxicity in neurodegenerative disease. EMBO Rep.

[10] Konopka A, Atkin JD (2022). The role of DNA damage in neural plasticity in physiology and neurodegeneration. Front Cell Neurosci.

[11] Simpson JE, Ince PG, Matthews FE, Shaw PJ, Heath PR, Brayne C, Garwood C, Higginbottom A, Wharton SB, Function MRCC, Ageing Neuropathology Study G (2015). A neuronal DNA damage response is detected at the earliest stages of Alzheimer's neuropathology and correlates with cognitive impairment in the Medical Research Council's Cognitive Function and Ageing Study ageing brain cohort. Neuropathol Appl Neurobiol.

[12] Zhang X, Heng Y, Kooistra SM, van Weering HRJ, Brummer ML, Gerrits E, Wesseling EM, Brouwer N, Nijboer TW, Dubbelaar ML, Boddeke E, Eggen BJL (2021). Intrinsic DNA damage repair deficiency results in progressive microglia loss and replacement. Glia.

[13] Vaidya A, Mao Z, Tian X, Spencer B, Seluanov A, Gorbunova V (2014). Knock-in reporter mice demonstrate that DNA repair by non-homologous end joining declines with age. PLoS Genet.

[14] Pao P-C, Patnaik D, Watson LA, Gao F, Pan L, Wang J, Adaikkan C, Penney J, Cam HP, Huang W-C, Pantano L, Lee A, Nott A, Phan TX, Gjoneska E, Elmsaouri S, Haggarty SJ, Tsai L-H (2020). HDAC1 modulates OGG1-initiated oxidative DNA damage repair in the aging brain and Alzheimer’s disease. Nat Commun.

[15] Perez-Correa J, Tharmapalan V, Geiger H, Wagner W (2022) Epigenetic clocks for mice based on age-associated regions that are conserved between mouse strains and human. bioRxiv. 10.1101/2022.03.23.485470 10.3389/fcell.2022.902857 PMC9204067 35721486

[16] Bell JT, Tsai PC, Yang TP, Pidsley R, Nisbet J, Glass D, Mangino M, Zhai G, Zhang F, Valdes A, Shin SY, Dempster EL, Murray RM, Grundberg E, Hedman AK, Nica A, Small KS, Mu TC, Dermitzakis ET, McCarthy MI, Mill J, Spector TD, Deloukas P (2012). Epigenome-wide scans identify differentially methylated regions for age and age-related phenotypes in a healthy ageing population. PLoS Genet.

[17] Johnson AA, Akman K, Calimport SR, Wuttke D, Stolzing A, de Magalhaes JP (2012). The role of DNA methylation in aging, rejuvenation, and age-related disease. Rejuvenation Res.

[18] Kass SU, Landsberger N, Wolffe AP (1997). DNA methylation directs a time-dependent repression of transcription initiation. Curr Biol.

[19] Sanchez-Mut JV, Heyn H, Vidal E, Moran S, Sayols S, Delgado-Morales R, Schultz MD, Ansoleaga B, Garcia-Esparcia P, Pons-Espinal M, de Lagran MM, Dopazo J, Rabano A, Avila J, Dierssen M, Lott I, Ferrer I, Ecker JR, Esteller M (2016). Human DNA methylomes of neurodegenerative diseases show common epigenomic patterns. Transl Psychiatry.

[20] Nabais MF, Laws SM, Lin T, Vallerga CL, Armstrong NJ, Blair IP, Kwok JB, Mather KA, Mellick GD, Sachdev PS, Wallace L, Henders AK, Zwamborn RAJ, Hop PJ, Lunnon K, Pishva E, Roubroeks JAY, Soininen H, Tsolaki M, Mecocci P, Lovestone S, Kloszewska I, Vellas B, Furlong S, Garton FC, Henderson RD, Mathers S, McCombe PA, Needham M, Ngo ST, Nicholson G, Pamphlett R, Rowe DB, Steyn FJ, Williams KL, Anderson TJ, Bentley SR, Dalrymple-Alford J, Fowder J, Gratten J, Halliday G, Hickie IB, Kennedy M, Lewis SJG, Montgomery GW, Pearson J, Pitcher TL, Silburn P, Zhang F, Visscher PM, Yang J, Stevenson AJ, Hillary RF, Marioni RE, Harris SE, Deary IJ, Jones AR, Shatunov A, Iacoangeli A, van Rheenen W, van den Berg LH, Shaw PJ, Shaw CE, Morrison KE, Al-Chalabi A, Veldink JH, Hannon E, Mill J, Wray NR, McRae AF, Australian Imaging B, Lifestyle s, Alzheimer's Disease Neuroimaging I (2021). Meta-analysis of genome-wide DNA methylation identifies shared associations across neurodegenerative disorders. Genome Biol.

[21] Mastroeni D, McKee A, Grover A, Rogers J, Coleman PD (2009). Epigenetic differences in cortical neurons from a pair of monozygotic twins discordant for Alzheimer's disease. PLoS ONE.

[22] Shay JW, Wright WE (2019). Telomeres and telomerase: three decades of progress. Nat Rev Genet.

[23] Rossiello F, Jurk D, Passos JF, d'Adda di Fagagna F (2022). Telomere dysfunction in ageing and age-related diseases. Nat Cell Biol.

[24] Tchkonia T, Zhu Y, van Deursen J, Campisi J, Kirkland JL (2013). Cellular senescence and the senescent secretory phenotype: therapeutic opportunities. J Clin Invest.

[25] Flanary BE, Streit WJ (2004). Progressive telomere shortening occurs in cultured rat microglia, but not astrocytes. Glia.

[26] Spilsbury A, Miwa S, Attems J, Saretzki G (2015). The role of telomerase protein TERT in Alzheimer's disease and in tau-related pathology in vitro. J Neurosci.

[27] Fu W, Killen M, Culmsee C, Dhar S, Pandita TK, Mattson MP (2000). The catalytic subunit of telomerase is expressed in developing brain neurons and serves a cell survival-promoting function. J Mol Neurosci.

[28] Caporaso GL, Lim DA, Alvarez-Buylla A, Chao MV (2003). Telomerase activity in the subventricular zone of adult mice. Mol Cell Neurosci.

[29] Liu MY, Nemes A, Zhou QG (2018). The emerging roles for telomerase in the central nervous system. Front Mol Neurosci.

[30] Levstek T, Kozjek E, Dolzan V, Trebusak Podkrajsek K (2020). Telomere attrition in neurodegenerative disorders. Front Cell Neurosci.

[31] Shim HS, Horner JW, Wu CJ, Li J, Lan ZD, Jiang S, Xu X, Hsu WH, Zal T, Flores II, Deng P, Lin YT, Tsai LH, Wang YA, DePinho RA (2021). Telomerase reverse transcriptase preserves neuron survival and cognition in Alzheimer's disease models. Nat Aging.

[32] Wan T, Weir EJ, Johnson M, Korolchuk VI, Saretzki GC (2021). Increased telomerase improves motor function and alpha-synuclein pathology in a transgenic mouse model of Parkinson's disease associated with enhanced autophagy. Prog Neurobiol.

[33] Scheffold A, Holtman IR, Dieni S, Brouwer N, Katz SF, Jebaraj BM, Kahle PJ, Hengerer B, Lechel A, Stilgenbauer S, Boddeke EW, Eggen BJ, Rudolph KL, Biber K (2016). Telomere shortening leads to an acceleration of synucleinopathy and impaired microglia response in a genetic mouse model. Acta Neuropathol Commun.

[34] Whittemore K, Derevyanko A, Martinez P, Serrano R, Pumarola M, Bosch F, Blasco MA (2019). Telomerase gene therapy ameliorates the effects of neurodegeneration associated to short telomeres in mice. Aging (Albany NY).

[35] Safaiyan S, Kannaiyan N, Snaidero N, Brioschi S, Biber K, Yona S, Edinger AL, Jung S, Rossner MJ, Simons M (2016). Age-related myelin degradation burdens the clearance function of microglia during aging. Nat Neurosci.

[36] Espay AJ, Vizcarra JA, Marsili L, Lang AE, Simon DK, Merola A, Josephs KA, Fasano A, Morgante F, Savica R, Greenamyre JT, Cambi F, Yamasaki TR, Tanner CM, Gan-Or Z, Litvan I, Mata IF, Zabetian CP, Brundin P, Fernandez HH, Standaert DG, Kauffman MA, Schwarzschild MA, Sardi SP, Sherer T, Perry G, Leverenz JB (2019). Revisiting protein aggregation as pathogenic in sporadic Parkinson and Alzheimer diseases. Neurology.

[37] Zhu PJ, Khatiwada S, Cui Y, Reineke LC, Dooling SW, Kim JJ, Li W, Walter P, Costa-Mattioli M (2019). Activation of the ISR mediates the behavioral and neurophysiological abnormalities in Down syndrome. Science.

[38] Krukowski K, Nolan A, Frias ES, Boone M, Ureta G, Grue K, Paladini MS, Elizarraras E, Delgado L, Bernales S, Walter P, Rosi S (2020). Small molecule cognitive enhancer reverses age-related memory decline in mice. Elife.

[39] Kim HJ, Cho MH, Shim WH, Kim JK, Jeon EY, Kim DH, Yoon SY (2017). Deficient autophagy in microglia impairs synaptic pruning and causes social behavioral defects. Mol Psychiatry.

[40] Sakai M, Austin J, Witmer F, Trueb L (1969). Studies of corpora amylacea: I. Isolation and preliminary characterization by chemical and histochemical techniques. Arch Neurol.

[41] Hervonen A, Koistinaho J, Alho H, Helen P, Santer RM, Rapoport SI (1986). Age related heretogeneity of lipopigments in human sympathetic ganglia. Mech Ageing Dev.

[42] Kepchia D, Huang L, Dargusch R, Rissman RA, Shokhirev MN, Fischer W, Schubert D (2020). Diverse proteins aggregate in mild cognitive impairment and Alzheimer’s disease brain. Alzheimer's Res Therapy.

[43] Trigo D, Nadais A, da Cruz ESOAB (2019). Unravelling protein aggregation as an ageing related process or a neuropathological response. Ageing Res Rev.

[44] Vonsattel JP, Myers RH, Stevens TJ, Ferrante RJ, Bird ED, Richardson EP (1985). Neuropathological classification of Huntington's disease. J Neuropathol Exp Neurol.

[45] Kuemmerle S, Gutekunst CA, Klein AM, Li XJ, Li SH, Beal MF, Hersch SM, Ferrante RJ (1999). Huntington aggregates may not predict neuronal death in Huntington's disease. Ann Neurol.

[46] Vos MJ, Carra S, Kanon B, Bosveld F, Klauke K, Sibon OC, Kampinga HH (2016). Specific protein homeostatic functions of small heat-shock proteins increase lifespan. Aging Cell.

[47] Mark KA, Dumas KJ, Bhaumik D, Schilling B, Davis S, Oron TR, Sorensen DJ, Lucanic M, Brem RB, Melov S, Ramanathan A, Gibson BW, Lithgow GJ (2016). Vitamin D promotes protein homeostasis and longevity via the stress response pathway genes skn-1, ire-1, and xbp-1. Cell Rep.

[48] Heard DS, Tuttle CSL, Lautenschlager NT, Maier AB (2018). Repurposing proteostasis-modifying drugs to prevent or treat age-related dementia: a systematic review. Front Physiol.

[49] Swerdlow RH (2020). The mitochondrial hypothesis: dysfunction, bioenergetic defects, and the metabolic link to Alzheimer's disease. Int Rev Neurobiol.

[50] Han R, Liang J, Zhou B (2021). Glucose metabolic dysfunction in neurodegenerative diseases-new mechanistic insights and the potential of hypoxia as a prospective therapy targeting metabolic reprogramming. Int J Mol Sci.

[51] Wang Y, Xu E, Musich PR, Lin F (2019). Mitochondrial dysfunction in neurodegenerative diseases and the potential countermeasure. CNS Neurosci Ther.

[52] Minhas PS, Latif-Hernandez A, McReynolds MR, Durairaj AS, Wang Q, Rubin A, Joshi AU, He JQ, Gauba E, Liu L, Wang C, Linde M, Sugiura Y, Moon PK, Majeti R, Suematsu M, Mochly-Rosen D, Weissman IL, Longo FM, Rabinowitz JD, Andreasson KI (2021). Restoring metabolism of myeloid cells reverses cognitive decline in ageing. Nature.

[53] de Lucia C, Murphy T, Steves CJ, Dobson RJB, Proitsi P, Thuret S (2020). Lifestyle mediates the role of nutrient-sensing pathways in cognitive aging: cellular and epidemiological evidence. Commun Biol.

[54] Kioussis B, Tuttle CSL, Heard DS, Kennedy BK, Lautenschlager NT, Maier AB (2021). Targeting impaired nutrient sensing with repurposed therapeutics to prevent or treat age-related cognitive decline and dementia: a systematic review. Ageing Res Rev.

[55] Wang D, Pascual JM, Yang H, Engelstad K, Mao X, Cheng J, Yoo J, Noebels JL, De Vivo DC (2006). A mouse model for Glut-1 haploinsufficiency. Hum Mol Genet.

[56] Kapogiannis D, Mattson MP (2011). Disrupted energy metabolism and neuronal circuit dysfunction in cognitive impairment and Alzheimer's disease. Lancet Neurol.

[57] Gollihue JL, Norris CM (2020). Astrocyte mitochondria: central players and potential therapeutic targets for neurodegenerative diseases and injury. Ageing Res Rev.

[58] Ishii T, Takanashi Y, Sugita K, Miyazawa M, Yanagihara R, Yasuda K, Onouchi H, Kawabe N, Nakata M, Yamamoto Y, Hartman PS, Ishii N (2017). Endogenous reactive oxygen species cause astrocyte defects and neuronal dysfunctions in the hippocampus: a new model for aging brain. Aging Cell.

[59] Wang C, Fan L, Khawaja RR, Liu B, Zhan L, Kodama L, Chin M, Li Y, Le D, Zhou Y, Condello C, Grinberg LT, Seeley WW, Miller BL, Mok S-A, Gestwicki JE, Cuervo AM, Luo W, Gan L (2022). Microglial NF-κB drives tau spreading and toxicity in a mouse model of tauopathy. Nat Commun.

[60] Zaghloul N, Kurepa D, Bader MY, Nagy N, Ahmed MN (2020). Prophylactic inhibition of NF-kappaB expression in microglia leads to attenuation of hypoxic ischemic injury of the immature brain. J Neuroinflammation.

[61] Sams EC (2021). Oligodendrocytes in the aging brain. Neuronal Signal.

[62] Michaels NJ, Lemmon K, Plemel JR, Jensen SK, Mishra MK, Brown D, Rawji KS, Koch M, Yong VW (2020). Aging-exacerbated acute axon and myelin injury is associated with microglia-derived reactive oxygen species and is alleviated by the generic medication indapamide. J Neurosci.

[63] Yin F, Boveris A, Cadenas E (2014). Mitochondrial energy metabolism and redox signaling in brain aging and neurodegeneration. Antioxid Redox Signal.

[64] Kataoka K, Bilkei-Gorzo A, Nozaki C, Togo A, Nakamura K, Ohta K, Zimmer A, Asahi T (2020). Age-dependent alteration in mitochondrial dynamics and autophagy in hippocampal neuron of cannabinoid CB1 receptor-deficient mice. Brain Res Bull.

[65] Becanovic K, Asghar M, Gadawska I, Sachdeva S, Walker D, Lazarowski ER, Franciosi S, Park KHJ, Cote HCF, Leavitt BR (2021). Age-related mitochondrial alterations in brain and skeletal muscle of the YAC128 model of Huntington disease. NPJ Aging Mech Dis.

[66] Liguori I, Russo G, Curcio F, Bulli G, Aran L, Della-Morte D, Gargiulo G, Testa G, Cacciatore F, Bonaduce D, Abete P (2018). Oxidative stress, aging, and diseases. Clin Interv Aging.

[67] Trigo D, Nadais A, Carvalho A, Morgado B, Santos F, Pereira S, da Cruz ESOAB (2022). Mitochondria dysfunction and impaired response to oxidative stress promotes proteostasis disruption in aged human cells. Mitochondrion.

[68] Takihara Y, Inatani M, Eto K, Inoue T, Kreymerman A, Miyake S, Ueno S, Nagaya M, Nakanishi A, Iwao K, Takamura Y, Sakamoto H, Satoh K, Kondo M, Sakamoto T, Goldberg JL, Nabekura J, Tanihara H (2015). In vivo imaging of axonal transport of mitochondria in the diseased and aged mammalian CNS. Proc Natl Acad Sci.

[69] Metzner K, Darawsha O, Wang M, Gaur N, Cheng Y, Rodiger A, Frahm C, Witte OW, Perocchi F, Axer H, Grosskreutz J, Brill MS (2022). Age-dependent increase of cytoskeletal components in sensory axons in human skin. Front Cell Dev Biol.

[70] Lamoureux PL, O'Toole MR, Heidemann SR, Miller KE (2010). Slowing of axonal regeneration is correlated with increased axonal viscosity during aging. BMC Neurosci.

[71] Ondaro J, Hernandez-Eguiazu H, Garciandia-Arcelus M, Loera-Valencia R, Rodriguez-Gomez L, Jimenez-Zuniga A, Goikolea J, Rodriguez-Rodriguez P, Ruiz-Martinez J, Moreno F, Lopez de Munain A, Holt IJ, Gil-Bea FJ, Gerenu G (2022). Defects of nutrient signaling and autophagy in neurodegeneration. Front Cell Dev Biol.

[72] Pan H, Finkel T (2017). Key proteins and pathways that regulate lifespan. J Biol Chem.

[73] Sadria M, Layton AT (2021). Interactions among mTORC, AMPK and SIRT: a computational model for cell energy balance and metabolism. Cell Commun Signal.

[74] Zadik Z, Chalew SA, McCarter RJ, Meistas M, Kowarski AA (1985). The influence of age on the 24-hour integrated concentration of growth hormone in normal individuals. J Clin Endocrinol Metab.

[75] Gubbi S, Quipildor GF, Barzilai N, Huffman DM, Milman S (2018). 40 years of IGF1: IGF1: the Jekyll and Hyde of the aging brain. J Mol Endocrinol.

[76] Saxton RA, Sabatini DM (2017). mTOR signaling in growth, metabolism, and disease. Cell.

[77] Andrews MG, Subramanian L, Kriegstein AR (2020). mTOR signaling regulates the morphology and migration of outer radial glia in developing human cortex. Elife.

[78] Guillen C, Benito M (2018). mTORC1 overactivation as a key aging factor in the progression to type 2 diabetes mellitus. Front Endocrinol (Lausanne).

[79] O' Neill C,  (2013). PI3-kinase/Akt/mTOR signaling: impaired on/off switches in aging, cognitive decline and Alzheimer's disease. Exp Gerontol.

[80] Laplante M, Sabatini DM (2012). mTOR signaling in growth control and disease. Cell.

[81] Yang F, Chu X, Yin M, Liu X, Yuan H, Niu Y, Fu L (2014). mTOR and autophagy in normal brain aging and caloric restriction ameliorating age-related cognition deficits. Behav Brain Res.

[82] Naseer A, Mir SS, Takacs-Vellai K, Nazir A (2021). Sirtuins and autophagy in age-associated neurodegenerative diseases: lessons from the *C. elegans* model. Int J Mol Sci.

[83] Satoh A, Imai SI, Guarente L (2017). The brain, sirtuins, and ageing. Nat Rev Neurosci.

[84] Steiner JL, Murphy EA, McClellan JL, Carmichael MD, Davis JM (2011). Exercise training increases mitochondrial biogenesis in the brain. J Appl Physiol (1985).

[85] Grabowska W, Sikora E, Bielak-Zmijewska A (2017). Sirtuins, a promising target in slowing down the ageing process. Biogerontology.

[86] Wang Y, Liang X, Chen Y, Zhao X (2016). Screening SIRT1 activators from medicinal plants as bioactive compounds against oxidative damage in mitochondrial function. Oxid Med Cell Longev.

[87] van de Ven RAH, Santos D, Haigis MC (2017). Mitochondrial sirtuins and molecular mechanisms of aging. Trends Mol Med.

[88] Balasubramaniam A, Li G, Ramanathan A, Mwangi SM, Hart CM, Arbiser JL, Srinivasan S (2022). SIRT3 activation promotes enteric neurons survival and differentiation. Sci Rep.

[89] Allen AR, Jones A, LoBianco FV, Krager KJ, Aykin-Burns N (2023). Effect of Sirt3 on hippocampal MnSOD activity, mitochondrial function, physiology, and cognition in an aged murine model. Behav Brain Res.

[90] Weir HJ, Yao P, Huynh FK, Escoubas CC, Goncalves RL, Burkewitz K, Laboy R, Hirschey MD, Mair WB (2017). Dietary restriction and AMPK increase lifespan via mitochondrial network and peroxisome remodeling. Cell Metab.

[91] Miranda M, Morici JF, Zanoni MB, Bekinschtein P (2019). Brain-derived neurotrophic factor: a key molecule for memory in the healthy and the pathological brain. Front Cell Neurosci.

[92] Zhao M, Cheng X, Lin X, Han Y, Zhou Y, Zhao T, He Y, Wu L, Zhao Y, Fan M, Zhu L (2019). Metformin administration prevents memory impairment induced by hypobaric hypoxia in rats. Behav Brain Res.

[93] Kumari R, Jat P (2021). Mechanisms of cellular senescence: cell cycle arrest and senescence associated secretory phenotype. Front Cell Dev Biol.

[94] Jeyapalan JC, Sedivy JM (2008). Cellular senescence and organismal aging. Mech Ageing Dev.

[95] Lopes-Paciencia S, Saint-Germain E, Rowell MC, Ruiz AF, Kalegari P, Ferbeyre G (2019). The senescence-associated secretory phenotype and its regulation. Cytokine.

[96] Birch J, Gil J (2020). Senescence and the SASP: many therapeutic avenues. Genes Dev.

[97] Baker DJ, Childs BG, Durik M, Wijers ME, Sieben CJ, Zhong J, Saltness RA, Jeganathan KB, Verzosa GC, Pezeshki A, Khazaie K, Miller JD, van Deursen JM (2016). Naturally occurring p16(Ink4a)-positive cells shorten healthy lifespan. Nature.

[98] Baker DJ, Wijshake T, Tchkonia T, LeBrasseur NK, Childs BG, van de Sluis B, Kirkland JL, van Deursen JM (2011). Clearance of p16Ink4a-positive senescent cells delays ageing-associated disorders. Nature.

[99] Sikora E, Bielak-Zmijewska A, Dudkowska M, Krzystyniak A, Mosieniak G, Wesierska M, Wlodarczyk J (2021). Cellular senescence in brain aging. Front Aging Neurosci.

[100] Dehkordi SK, Walker J, Sah E, Bennett E, Atrian F, Frost B, Woost B, Bennett RE, Orr TC, Zhou Y, Andhey PS, Colonna M, Sudmant PH, Xu P, Wang M, Zhang B, Zare H, Orr ME (2021). Profiling senescent cells in human brains reveals neurons with CDKN2D/p19 and tau neuropathology. Nat Aging.

[101] Musi N, Valentine JM, Sickora KR, Baeuerle E, Thompson CS, Shen Q, Orr ME (2018). Tau protein aggregation is associated with cellular senescence in the brain. Aging Cell.

[102] Riessland M, Kolisnyk B, Kim TW, Cheng J, Ni J, Pearson JA, Park EJ, Dam K, Acehan D, Ramos-Espiritu LS, Wang W, Zhang J, Shim JW, Ciceri G, Brichta L, Studer L, Greengard P (2019). Loss of SATB1 induces p21-dependent cellular senescence in post-mitotic dopaminergic neurons. Cell Stem Cell.

[103] Fatt MP, Tran LM, Vetere G, Storer MA, Simonetta JV, Miller FD, Frankland PW, Kaplan DR (2022). Restoration of hippocampal neural precursor function by ablation of senescent cells in the aging stem cell niche. Stem Cell Rep.

[104] Zhang P, Kishimoto Y, Grammatikakis I, Gottimukkala K, Cutler RG, Zhang S, Abdelmohsen K, Bohr VA, Misra Sen J, Gorospe M, Mattson MP (2019). Senolytic therapy alleviates Abeta-associated oligodendrocyte progenitor cell senescence and cognitive deficits in an Alzheimer's disease model. Nat Neurosci.

[105] Gaikwad S, Puangmalai N, Bittar A, Montalbano M, Garcia S, McAllen S, Bhatt N, Sonawane M, Sengupta U, Kayed R (2021). Tau oligomer induced HMGB1 release contributes to cellular senescence and neuropathology linked to Alzheimer's disease and frontotemporal dementia. Cell Rep.

[106] Chinta SJ, Woods G, Demaria M, Rane A, Zou Y, McQuade A, Rajagopalan S, Limbad C, Madden DT, Campisi J, Andersen JK (2018). Cellular senescence is induced by the environmental neurotoxin Paraquat and contributes to neuropathology linked to Parkinson's disease. Cell Rep.

[107] Verma DK, Seo BA, Ghosh A, Ma SX, Hernandez-Quijada K, Andersen JK, Ko HS, Kim YH (2021). Alpha-synuclein preformed fibrils induce cellular senescence in Parkinson's disease models. Cells.

[108] Yoon YS, You JS, Kim TK, Ahn WJ, Kim MJ, Son KH, Ricarte D, Ortiz D, Lee SJ, Lee HJ (2022). Senescence and impaired DNA damage responses in alpha-synucleinopathy models. Exp Mol Med.

[109] Fielder E, Wan T, Alimohammadiha G, Ishaq A, Low E, Weigand BM, Kelly G, Parker C, Griffin B, Jurk D, Korolchuk VI, von Zglinicki T, Miwa S (2022). Short senolytic or senostatic interventions rescue progression of radiation-induced frailty and premature ageing in mice. Elife.

[110] Krzystyniak A, Wesierska M, Petrazzo G, Gadecka A, Dudkowska M, Bielak-Zmijewska A, Mosieniak G, Figiel I, Wlodarczyk J, Sikora E (2022). Combination of dasatinib and quercetin improves cognitive abilities in aged male Wistar rats, alleviates inflammation and changes hippocampal synaptic plasticity and histone H3 methylation profile. Aging (Albany NY).

[111] Bussian TJ, Aziz A, Meyer CF, Swenson BL, van Deursen JM, Baker DJ (2018). Clearance of senescent glial cells prevents tau-dependent pathology and cognitive decline. Nature.

[112] Navarro Negredo P, Yeo RW, Brunet A (2020). Aging and rejuvenation of neural stem cells and their niches. Cell Stem Cell.

[113] Nicaise AM, Willis CM, Crocker SJ, Pluchino S (2020). Stem cells of the aging brain. Front Aging Neurosci.

[114] Ibrayeva A, Bay M, Pu E, Jorg DJ, Peng L, Jun H, Zhang N, Aaron D, Lin C, Resler G, Hidalgo A, Jang MH, Simons BD, Bonaguidi MA (2021). Early stem cell aging in the mature brain. Cell Stem Cell.

[115] Ben Abdallah NM, Slomianka L, Vyssotski AL, Lipp HP (2010). Early age-related changes in adult hippocampal neurogenesis in C57 mice. Neurobiol Aging.

[116] Kempermann G, Gage FH, Aigner L, Song H, Curtis MA, Thuret S, Kuhn HG, Jessberger S, Frankland PW, Cameron HA, Gould E, Hen R, Abrous DN, Toni N, Schinder AF, Zhao X, Lucassen PJ, Frisen J (2018). Human adult neurogenesis: evidence and remaining questions. Cell Stem Cell.

[117] Sorrells SF, Paredes MF, Cebrian-Silla A, Sandoval K, Qi D, Kelley KW, James D, Mayer S, Chang J, Auguste KI, Chang EF, Gutierrez AJ, Kriegstein AR, Mathern GW, Oldham MC, Huang EJ, Garcia-Verdugo JM, Yang Z, Alvarez-Buylla A (2018). Human hippocampal neurogenesis drops sharply in children to undetectable levels in adults. Nature.

[118] Boldrini M, Fulmore CA, Tartt AN, Simeon LR, Pavlova I, Poposka V, Rosoklija GB, Stankov A, Arango V, Dwork AJ, Hen R, Mann JJ (2018). Human hippocampal neurogenesis persists throughout aging. Cell Stem Cell.

[119] Li YD, Luo YJ, Chen ZK, Quintanilla L, Cherasse Y, Zhang L, Lazarus M, Huang ZL, Song J (2022). Hypothalamic modulation of adult hippocampal neurogenesis in mice confers activity-dependent regulation of memory and anxiety-like behavior. Nat Neurosci.

[120] Moreno-Jimenez EP, Flor-Garcia M, Terreros-Roncal J, Rabano A, Cafini F, Pallas-Bazarra N, Avila J, Llorens-Martin M (2019). Adult hippocampal neurogenesis is abundant in neurologically healthy subjects and drops sharply in patients with Alzheimer's disease. Nat Med.

[121] Tobin MK, Musaraca K, Disouky A, Shetti A, Bheri A, Honer WG, Kim N, Dawe RJ, Bennett DA, Arfanakis K, Lazarov O (2019). Human hippocampal neurogenesis persists in aged adults and Alzheimer's disease patients. Cell Stem Cell.

[122] Li Puma DD, Piacentini R, Grassi C (2020). Does impairment of adult neurogenesis contribute to pathophysiology of Alzheimer's disease? A still open question. Front Mol Neurosci.

[123] Trivino JJ, von Bernhardi R (2021). The effect of aged microglia on synaptic impairment and its relevance in neurodegenerative diseases. Neurochem Int.

[124] Stephenson J, Nutma E, van der Valk P, Amor S (2018). Inflammation in CNS neurodegenerative diseases. Immunology.

[125] Magalhaes J, Tresse E, Ejlerskov P, Hu E, Liu Y, Marin A, Montalant A, Satriano L, Rundsten CF, Carlsen EMM, Rydbirk R, Sharifi-Zarchi A, Andersen JB, Aznar S, Brudek T, Khodosevich K, Prinz M, Perrier JM, Sharma M, Gasser T, Issazadeh-Navikas S (2021). PIAS2-mediated blockade of IFN-beta signaling: a basis for sporadic Parkinson disease dementia. Mol Psychiatry.

[126] Silva I, Silva J, Ferreira R, Trigo D (2021). Glymphatic system, AQP4, and their implications in Alzheimer's disease. Neurol Res Pract.

[127] Gordleeva S, Kanakov O, Ivanchenko M, Zaikin A, Franceschi C (2020). Brain aging and garbage cleaning: modelling the role of sleep, glymphatic system, and microglia senescence in the propagation of inflammaging. Semin Immunopathol.

[128] Kress BT, Iliff JJ, Xia M, Wang M, Wei HS, Zeppenfeld D, Xie L, Kang H, Xu Q, Liew JA, Plog BA, Ding F, Deane R, Nedergaard M (2014). Impairment of paravascular clearance pathways in the aging brain. Ann Neurol.

[129] Zhou Y, Cai J, Zhang W, Gong X, Yan S, Zhang K, Luo Z, Sun J, Jiang Q, Lou M (2020). Impairment of the glymphatic pathway and putative meningeal lymphatic vessels in the aging human. Ann Neurol.

[130] Kaur J, Fahmy LM, Davoodi-Bojd E, Zhang L, Ding G, Hu J, Zhang Z, Chopp M, Jiang Q (2021). Waste clearance in the brain. Front Neuroanat.

[131] Villeda SA, Luo J, Mosher KI, Zou B, Britschgi M, Bieri G, Stan TM, Fainberg N, Ding Z, Eggel A, Lucin KM, Czirr E, Park JS, Couillard-Despres S, Aigner L, Li G, Peskind ER, Kaye JA, Quinn JF, Galasko DR, Xie XS, Rando TA, Wyss-Coray T (2011). The ageing systemic milieu negatively regulates neurogenesis and cognitive function. Nature.

[132] Ouanounou A, Zhang L, Charlton MP, Carlen PL (1999). Differential modulation of synaptic transmission by calcium chelators in young and aged hippocampal CA1 neurons: evidence for altered calcium homeostasis in aging. J Neurosci.

[133] Chandran R, Kumar M, Kesavan L, Jacob RS, Gunasekaran S, Lakshmi S, Sadasivan C, Omkumar RV (2019). Cellular calcium signaling in the aging brain. J Chem Neuroanat.

[134] Toescu EC, Verkhratsky A (2007). The importance of being subtle: small changes in calcium homeostasis control cognitive decline in normal aging. Aging Cell.

[135] Gant JC, Chen KC, Kadish I, Blalock EM, Thibault O, Porter NM, Landfield PW (2015). Reversal of aging-related neuronal Ca^2+^ dysregulation and cognitive impairment by delivery of a transgene encoding FK506-binding protein 126/1b to the hippocampus. J Neurosci.

[136] Blaszczyk JW (2020). Energy metabolism decline in the aging brain-pathogenesis of neurodegenerative disorders. Metabolites.

[137] Franzoni F, Scarfo G, Guidotti S, Fusi J, Asomov M, Pruneti C (2021). Oxidative stress and cognitive decline: the neuroprotective role of natural antioxidants. Front Neurosci.

[138] Tse KH, Herrup K (2017). DNA damage in the oligodendrocyte lineage and its role in brain aging. Mech Ageing Dev.

[139] Filley CM, Fields RD (2016). White matter and cognition: making the connection. J Neurophysiol.

[140] Belsky DW, Caspi A, Houts R, Cohen HJ, Corcoran DL, Danese A, Harrington H, Israel S, Levine ME, Schaefer JD, Sugden K, Williams B, Yashin AI, Poulton R, Moffitt TE (2015). Quantification of biological aging in young adults. Proc Natl Acad Sci USA.

[141] Nie C, Li Y, Li R, Yan Y, Zhang D, Li T, Li Z, Sun Y, Zhen H, Ding J, Wan Z, Gong J, Shi Y, Huang Z, Wu Y, Cai K, Zong Y, Wang Z, Wang R, Jian M, Jin X, Wang J, Yang H, Han JJ, Zhang X, Franceschi C, Kennedy BK, Xu X (2022). Distinct biological ages of organs and systems identified from a multi-omics study. Cell Rep.

[142] Schaum N, Lehallier B, Hahn O, Palovics R, Hosseinzadeh S, Lee SE, Sit R, Lee DP, Losada PM, Zardeneta ME, Fehlmann T, Webber JT, McGeever A, Calcuttawala K, Zhang H, Berdnik D, Mathur V, Tan W, Zee A, Tan M, Tabula Muris C, Pisco AO, Karkanias J, Neff NF, Keller A, Darmanis S, Quake SR, Wyss-Coray T (2020). Ageing hallmarks exhibit organ-specific temporal signatures. Nature.

[143] Pandya VA, Patani R (2021). Region-specific vulnerability in neurodegeneration: lessons from normal ageing. Ageing Res Rev.

[144] Bender AR, Volkle MC, Raz N (2016). Differential aging of cerebral white matter in middle-aged and older adults: a seven-year follow-up. Neuroimage.

[145] Dotson VM, Szymkowicz SM, Sozda CN, Kirton JW, Green ML, O'Shea A, McLaren ME, Anton SD, Manini TM, Woods AJ (2015). Age differences in prefrontal surface area and thickness in middle aged to older adults. Front Aging Neurosci.

[146] Bettio LEB, Rajendran L, Gil-Mohapel J (2017). The effects of aging in the hippocampus and cognitive decline. Neurosci Biobehav Rev.

[147] Mattson MP, Magnus T (2006). Ageing and neuronal vulnerability. Nat Rev Neurosci.

[148] Wang X, Michaelis EK (2010). Selective neuronal vulnerability to oxidative stress in the brain. Front Aging Neurosci.

[149] Soreq L, Rose J, Soreq E, Hardy J, Trabzuni D, Cookson MR, Smith C, Ryten M, Patani R, Ule J, Consortium UKBE, North American Brain Expression C (2017). Major shifts in glial regional identity are a transcriptional hallmark of human brain aging. Cell Rep.

[150] Jobson DD, Hase Y, Clarkson AN, Kalaria RN (2021). The role of the medial prefrontal cortex in cognition, ageing and dementia. Brain Commun.

[151] Smith DE, Rapp PR, McKay HM, Roberts JA, Tuszynski MH (2004). Memory impairment in aged primates is associated with focal death of cortical neurons and atrophy of subcortical neurons. J Neurosci.

[152] Morrison JH, Baxter MG (2012). The ageing cortical synapse: hallmarks and implications for cognitive decline. Nat Rev Neurosci.

[153] Wruck W, Adjaye J (2020). Meta-analysis of human prefrontal cortex reveals activation of GFAP and decline of synaptic transmission in the aging brain. Acta Neuropathol Commun.

[154] Chan TE, Grossman YS, Bloss EB, Janssen WG, Lou W, McEwen BS, Dumitriu D, Morrison JH (2018). Cell-type specific changes in glial morphology and glucocorticoid expression during stress and aging in the medial prefrontal cortex. Front Aging Neurosci.

[155] Ximerakis M, Lipnick SL, Innes BT, Simmons SK, Adiconis X, Dionne D, Mayweather BA, Nguyen L, Niziolek Z, Ozek C, Butty VL, Isserlin R, Buchanan SM, Levine SS, Regev A, Bader GD, Levin JZ, Rubin LL (2019). Single-cell transcriptomic profiling of the aging mouse brain. Nat Neurosci.

[156] Allen WE, Blosser TR, Sullivan ZA, Dulac C, Zhuang X (2022). Molecular and spatial signatures of mouse brain aging at single-cell resolution. Cell.

[157] Chen ZH, Li S, Xu M, Liu CC, Ye H, Wang B, Wu QF (2022). Single-cell transcriptomic profiling of the hypothalamic median eminence during aging. J Genet Genomics.

[158] Luquez T, Gaur P, Kosater IM, Lam M, Lee DI, Mares J, Paryani F, Yadav A, Menon V (2022). Cell type-specific changes identified by single-cell transcriptomics in Alzheimer's disease. Genome Med.

[159] Buckley MT, Sun ED, George BM, Liu L, Schaum N, Xu L, Reyes JM, Goodell MA, Weissman IL, Wyss-Coray T, Rando TA, Brunet A (2022). Cell-type-specific aging clocks to quantify aging and rejuvenation in neurogenic regions of the brain. Nature Aging.

[160] Villeda SA, Plambeck KE, Middeldorp J, Castellano JM, Mosher KI, Luo J, Smith LK, Bieri G, Lin K, Berdnik D, Wabl R, Udeochu J, Wheatley EG, Zou B, Simmons DA, Xie XS, Longo FM, Wyss-Coray T (2014). Young blood reverses age-related impairments in cognitive function and synaptic plasticity in mice. Nat Med.

[161] Katsimpardi L, Litterman NK, Schein PA, Miller CM, Loffredo FS, Wojtkiewicz GR, Chen JW, Lee RT, Wagers AJ, Rubin LL (2014). Vascular and neurogenic rejuvenation of the aging mouse brain by young systemic factors. Science.

[162] Das MM, Godoy M, Chen S, Moser VA, Avalos P, Roxas KM, Dang I, Yanez A, Zhang W, Bresee C, Arditi M, Liu GY, Svendsen CN, Goodridge HS (2019). Young bone marrow transplantation preserves learning and memory in old mice. Commun Biol.

[163] Ogrodnik M, Evans SA, Fielder E, Victorelli S, Kruger P, Salmonowicz H, Weigand BM, Patel AD, Pirtskhalava T, Inman CL, Johnson KO, Dickinson SL, Rocha A, Schafer MJ, Zhu Y, Allison DB, von Zglinicki T, LeBrasseur NK, Tchkonia T, Neretti N, Passos JF, Kirkland JL, Jurk D (2021). Whole-body senescent cell clearance alleviates age-related brain inflammation and cognitive impairment in mice. Aging Cell.

[164] Pereira T, Cipriano I, Costa T, Saraiva M, Martins A, Consortium AGl (2019). Exercise, ageing and cognitive function—effects of a personalized physical exercise program in the cognitive function of older adults. Physiol Behav.

[165] Garcia-Matas S, Paul RK, Molina-Martinez P, Palacios H, Gutierrez VM, Corpas R, Pallas M, Cristofol R, de Cabo R, Sanfeliu C (2015). In vitro caloric restriction induces protective genes and functional rejuvenation in senescent SAMP8 astrocytes. Aging Cell.

[166] Lin T, Liu GA, Perez E, Rainer RD, Febo M, Cruz-Almeida Y, Ebner NC (2018). Systemic inflammation mediates age-related cognitive deficits. Front Aging Neurosci.

[167] Fielder E, Tweedy C, Wilson C, Oakley F, LeBeau FEN, Passos JF, Mann DA, von Zglinicki T, Jurk D (2020). Anti-inflammatory treatment rescues memory deficits during aging in nfkb1(−/−) mice. Aging Cell.

[168] Lutshumba J, Nikolajczyk BS, Bachstetter AD (2021). Dysregulation of systemic immunity in aging and dementia. Front Cell Neurosci.

[169] Franceschi C, Garagnani P, Parini P, Giuliani C, Santoro A (2018). Inflammaging: a new immune-metabolic viewpoint for age-related diseases. Nat Rev Endocrinol.

[170] Rutsch A, Kantsjo JB, Ronchi F (2020). The gut-brain axis: how microbiota and host inflammasome influence brain physiology and pathology. Front Immunol.

[171] Mou Y, Du Y, Zhou L, Yue J, Hu X, Liu Y, Chen S, Lin X, Zhang G, Xiao H, Dong B (2022). Gut microbiota interact with the brain through systemic chronic inflammation: implications on neuroinflammation, neurodegeneration, and aging. Front Immunol.

[172] Daneman R, Prat A (2015). The blood–brain barrier. Cold Spring Harb Perspect Biol.

[173] Knox EG, Aburto MR, Clarke G, Cryan JF, O'Driscoll CM (2022). The blood–brain barrier in aging and neurodegeneration. Mol Psychiatry.

[174] Jansson D, Rustenhoven J, Feng S, Hurley D, Oldfield RL, Bergin PS, Mee EW, Faull RL, Dragunow M (2014). A role for human brain pericytes in neuroinflammation. J Neuroinflammation.

[175] Iram T, Kern F, Kaur A, Myneni S, Morningstar AR, Shin H, Garcia MA, Yerra L, Palovics R, Yang AC, Hahn O, Lu N, Shuken SR, Haney MS, Lehallier B, Iyer M, Luo J, Zetterberg H, Keller A, Zuchero JB, Wyss-Coray T (2022). Young CSF restores oligodendrogenesis and memory in aged mice via Fgf17. Nature.

[176] Rege SV, Teichert A, Masumi J, Dhande OS, Harish R, Higgins BW, Lopez Y, Akrapongpisak L, Hackbart H, Caryotakis S, Leone DP, Szoke B, Hannestad J, Nikolich K, Braithwaite SP, Minami SS (2023). CCR3 plays a role in murine age-related cognitive changes and T-cell infiltration into the brain. Commun Biol.

[177] Horvath S, Gurven M, Levine ME, Trumble BC, Kaplan H, Allayee H, Ritz BR, Chen B, Lu AT, Rickabaugh TM, Jamieson BD, Sun D, Li S, Chen W, Quintana-Murci L, Fagny M, Kobor MS, Tsao PS, Reiner AP, Edlefsen KL, Absher D, Assimes TL (2016). An epigenetic clock analysis of race/ethnicity, sex, and coronary heart disease. Genome Biol.

[178] Goyal MS, Blazey TM, Su Y, Couture LE, Durbin TJ, Bateman RJ, Benzinger TL, Morris JC, Raichle ME, Vlassenko AG (2019). Persistent metabolic youth in the aging female brain. Proc Natl Acad Sci USA.

[179] Berchtold NC, Cribbs DH, Coleman PD, Rogers J, Head E, Kim R, Beach T, Miller C, Troncoso J, Trojanowski JQ, Zielke HR, Cotman CW (2008). Gene expression changes in the course of normal brain aging are sexually dimorphic. Proc Natl Acad Sci USA.

[180] Castellano JM, Mosher KI, Abbey RJ, McBride AA, James ML, Berdnik D, Shen JC, Zou B, Xie XS, Tingle M, Hinkson IV, Angst MS, Wyss-Coray T (2017). Human umbilical cord plasma proteins revitalize hippocampal function in aged mice. Nature.

[181] Smith LK, Verovskaya E, Bieri G, Horowitz AM, von Ungern-Sternberg SNI, Lin K, Seizer P, Passegue E, Villeda SA (2020). The aged hematopoietic system promotes hippocampal-dependent cognitive decline. Aging Cell.

[182] Khrimian L, Obri A, Ramos-Brossier M, Rousseaud A, Moriceau S, Nicot AS, Mera P, Kosmidis S, Karnavas T, Saudou F, Gao XB, Oury F, Kandel E, Karsenty G (2017). Gpr158 mediates osteocalcin's regulation of cognition. J Exp Med.

[183] Gan KJ, Sudhof TC (2019). Specific factors in blood from young but not old mice directly promote synapse formation and NMDA-receptor recruitment. Proc Natl Acad Sci USA.

[184] Yousef H, Czupalla CJ, Lee D, Chen MB, Burke AN, Zera KA, Zandstra J, Berber E, Lehallier B, Mathur V, Nair RV, Bonanno LN, Yang AC, Peterson T, Hadeiba H, Merkel T, Korbelin J, Schwaninger M, Buckwalter MS, Quake SR, Butcher EC, Wyss-Coray T (2019). Aged blood impairs hippocampal neural precursor activity and activates microglia via brain endothelial cell VCAM1. Nat Med.

[185] van Praag H, Shubert T, Zhao C, Gage FH (2005). Exercise enhances learning and hippocampal neurogenesis in aged mice. J Neurosci.

[186] Voss MW, Soto C, Yoo S, Sodoma M, Vivar C, van Praag H (2019). Exercise and hippocampal memory systems. Trends Cogn Sci.

[187] Pluvinage JV, Wyss-Coray T (2020). Systemic factors as mediators of brain homeostasis, ageing and neurodegeneration. Nat Rev Neurosci.

[188] Morland C, Andersson KA, Haugen OP, Hadzic A, Kleppa L, Gille A, Rinholm JE, Palibrk V, Diget EH, Kennedy LH, Stolen T, Hennestad E, Moldestad O, Cai Y, Puchades M, Offermanns S, Vervaeke K, Bjoras M, Wisloff U, Storm-Mathisen J, Bergersen LH (2017). Exercise induces cerebral VEGF and angiogenesis via the lactate receptor HCAR1. Nat Commun.

[189] Fabel K, Fabel K, Tam B, Kaufer D, Baiker A, Simmons N, Kuo CJ, Palmer TD (2003). VEGF is necessary for exercise-induced adult hippocampal neurogenesis. Eur J Neurosci.

[190] Sleiman SF, Henry J, Al-Haddad R, El Hayek L, Abou Haidar E, Stringer T, Ulja D, Karuppagounder SS, Holson EB, Ratan RR, Ninan I, Chao MV (2016). Exercise promotes the expression of brain derived neurotrophic factor (BDNF) through the action of the ketone body beta-hydroxybutyrate. Elife.

[191] Horowitz AM, Fan X, Bieri G, Smith LK, Sanchez-Diaz CI, Schroer AB, Gontier G, Casaletto KB, Kramer JH, Williams KE, Villeda SA (2020). Blood factors transfer beneficial effects of exercise on neurogenesis and cognition to the aged brain. Science.

[192] Andreotti DZ, Silva JDN, Matumoto AM, Orellana AM, de Mello PS, Kawamoto EM (2020). Effects of physical exercise on autophagy and apoptosis in aged brain: human and animal studies. Front Nutr.

[193] Ross JM, Coppotelli G, Branca RM, Kim KM, Lehtio J, Sinclair DA, Olson L (2019). Voluntary exercise normalizes the proteomic landscape in muscle and brain and improves the phenotype of progeroid mice. Aging Cell.

[194] Lin TW, Kuo YM (2013). Exercise benefits brain function: the monoamine connection. Brain Sci.

[195] Vecchio LM, Meng Y, Xhima K, Lipsman N, Hamani C, Aubert I (2018). The neuroprotective effects of exercise: maintaining a healthy brain throughout aging. Brain Plast.

[196] Fernandes J, Arida RM, Gomez-Pinilla F (2017). Physical exercise as an epigenetic modulator of brain plasticity and cognition. Neurosci Biobehav Rev.

[197] Masoro EJ (2005). Overview of caloric restriction and ageing. Mech Ageing Dev.

[198] Flanagan EW, Most J, Mey JT, Redman LM (2020). Calorie restriction and aging in humans. Annu Rev Nutr.

[199] Bouchard J, Villeda SA (2015). Aging and brain rejuvenation as systemic events. J Neurochem.

[200] Gredilla R, Barja G (2005). Minireview: the role of oxidative stress in relation to caloric restriction and longevity. Endocrinology.

[201] Heydari AR, Unnikrishnan A, Lucente LV, Richardson A (2007). Caloric restriction and genomic stability. Nucleic Acids Res.

[202] Asami T, Endo K, Matsui R, Sawa T, Tanaka Y, Saiki T, Tanba N, Haga H, Tanaka S (2022). Long-term caloric restriction ameliorates T cell immunosenescence in mice. Mech Ageing Dev.

[203] Spadaro O, Youm Y, Shchukina I, Ryu S, Sidorov S, Ravussin A, Nguyen K, Aladyeva E, Predeus AN, Smith SR, Ravussin E, Galban C, Artyomov MN, Dixit VD (2022). Caloric restriction in humans reveals immunometabolic regulators of health span. Science.

[204] Cerletti M, Jang YC, Finley LW, Haigis MC, Wagers AJ (2012). Short-term calorie restriction enhances skeletal muscle stem cell function. Cell Stem Cell.

[205] Yilmaz OH, Katajisto P, Lamming DW, Gultekin Y, Bauer-Rowe KE, Sengupta S, Birsoy K, Dursun A, Yilmaz VO, Selig M, Nielsen GP, Mino-Kenudson M, Zukerberg LR, Bhan AK, Deshpande V, Sabatini DM (2012). mTORC1 in the Paneth cell niche couples intestinal stem-cell function to calorie intake. Nature.

[206] Mattson MP (2010). The impact of dietary energy intake on cognitive aging. Front Aging Neurosci.

[207] Apple DM, Mahesula S, Fonseca RS, Zhu C, Kokovay E (2019). Calorie restriction protects neural stem cells from age-related deficits in the subventricular zone. Aging (Albany NY).

[208] Hyun DH, Emerson SS, Jo DG, Mattson MP, de Cabo R (2006). Calorie restriction up-regulates the plasma membrane redox system in brain cells and suppresses oxidative stress during aging. Proc Natl Acad Sci USA.

[209] Zhang L, Xu H, Ding N, Li X, Chen X, Chen Z (2021). Beneficial effects on brain micro-environment by caloric restriction in alleviating neurodegenerative diseases and brain aging. Front Physiol.

[210] Allison J, Kaliszewska A, Uceda S, Reiriz M, Arias N (2021). Targeting DNA methylation in the adult brain through diet. Nutrients.

[211] Kim EC, Kim JR (2019). Senotherapeutics: emerging strategy for healthy aging and age-related disease. BMB Rep.

[212] Lee S, Wang EY, Steinberg AB, Walton CC, Chinta SJ, Andersen JK (2021). A guide to senolytic intervention in neurodegenerative disease. Mech Ageing Dev.

[213] Zhang X, Pearsall VM, Carver CM, Atkinson EJ, Clarkson BDS, Grund EM, Baez-Faria M, Pavelko KD, Kachergus JM, White TA, Johnson RK, Malo CS, Gonzalez-Suarez AM, Ayasoufi K, Johnson KO, Tritz ZP, Fain CE, Khadka RH, Ogrodnik M, Jurk D, Zhu Y, Tchkonia T, Revzin A, Kirkland JL, Johnson AJ, Howe CL, Thompson EA, LeBrasseur NK, Schafer MJ (2022). Rejuvenation of the aged brain immune cell landscape in mice through p16-positive senescent cell clearance. Nat Commun.

[214] Yousefzadeh MJ, Wilkinson JE, Hughes B, Gadela N, Ladiges WC, Vo N, Niedernhofer LJ, Huffman DM, Robbins PD (2020). Heterochronic parabiosis regulates the extent of cellular senescence in multiple tissues. Geroscience.

[215] Mehdipour M, Mehdipour T, Skinner CM, Wong N, Liu C, Chen CC, Jeon OH, Zuo Y, Conboy MJ, Conboy IM (2021). Plasma dilution improves cognition and attenuates neuroinflammation in old mice. Geroscience.

[216] Da Silva-Alvarez S, Guerra-Varela J, Sobrido-Camean D, Quelle A, Barreiro-Iglesias A, Sanchez L, Collado M (2020). Cell senescence contributes to tissue regeneration in zebrafish. Aging Cell.

[217] Munoz-Espin D, Canamero M, Maraver A, Gomez-Lopez G, Contreras J, Murillo-Cuesta S, Rodriguez-Baeza A, Varela-Nieto I, Ruberte J, Collado M, Serrano M (2013). Programmed cell senescence during mammalian embryonic development. Cell.

[218] Kang C (2019). Senolytics and senostatics: a two-pronged approach to target cellular senescence for delaying aging and age-related diseases. Mol Cells.

[219] Ribeiro R, Macedo JC, Costa M, Ustiyan V, Shindyapina AV, Tyshkovskiy A, Gomes RN, Castro JP, Kalin TV, Vasques-Nóvoa F, Nascimento DS, Dmitriev SE, Gladyshev VN, Kalinichenko VV, Logarinho E (2022). In vivo cyclic induction of the FOXM1 transcription factor delays natural and progeroid aging phenotypes and extends healthspan. Nat Aging.

[220] Takahashi K, Yamanaka S (2006). Induction of pluripotent stem cells from mouse embryonic and adult fibroblast cultures by defined factors. Cell.

[221] Yu J, Vodyanik MA, Smuga-Otto K, Antosiewicz-Bourget J, Frane JL, Tian S, Nie J, Jonsdottir GA, Ruotti V, Stewart R, Slukvin II, Thomson JA (2007). Induced pluripotent stem cell lines derived from human somatic cells. Science.

[222] Lapasset L, Milhavet O, Prieur A, Besnard E, Babled A, Ait-Hamou N, Leschik J, Pellestor F, Ramirez JM, De Vos J, Lehmann S, Lemaitre JM (2011). Rejuvenating senescent and centenarian human cells by reprogramming through the pluripotent state. Genes Dev.

[223] Alle Q, Le Borgne E, Milhavet O, Lemaitre JM (2021). Reprogramming: emerging strategies to rejuvenate aging cells and tissues. Int J Mol Sci.

[224] Alle Q, Le Borgne E, Bensadoun P, Lemey C, Bechir N, Gabanou M, Estermann F, Bertrand-Gaday C, Pessemesse L, Toupet K, Desprat R, Vialaret J, Hirtz C, Noel D, Jorgensen C, Casas F, Milhavet O, Lemaitre JM (2022). A single short reprogramming early in life initiates and propagates an epigenetically related mechanism improving fitness and promoting an increased healthy lifespan. Aging Cell.

[225] Rodriguez-Matellan A, Alcazar N, Hernandez F, Serrano M, Avila J (2020). In vivo reprogramming ameliorates aging features in dentate gyrus cells and improves memory in mice. Stem Cell Reports.

[226] Clark IH, Roman A, Fellows E, Radha S, Var SR, Roushdy Z, Borer SM, Johnson S, Chen O, Borgida JS, Steevens A, Shetty A, Strell P, Low WC, Grande AW (2022). Cell reprogramming for regeneration and repair of the nervous system. Biomedicines.

[227] Gomes AP, Price NL, Ling AJ, Moslehi JJ, Montgomery MK, Rajman L, White JP, Teodoro JS, Wrann CD, Hubbard BP, Mercken EM, Palmeira CM, de Cabo R, Rolo AP, Turner N, Bell EL, Sinclair DA (2013). Declining NAD(+) induces a pseudohypoxic state disrupting nuclear-mitochondrial communication during aging. Cell.

[228] Xie X, Gao Y, Zeng M, Wang Y, Wei TF, Lu YB, Zhang WP (2019). Nicotinamide ribose ameliorates cognitive impairment of aged and Alzheimer's disease model mice. Metab Brain Dis.

[229] Vreones M, Mustapic M, Moaddel R, Pucha KA, Lovett J, Seals DR, Kapogiannis D, Martens CR (2023). Oral nicotinamide riboside raises NAD+ and lowers biomarkers of neurodegenerative pathology in plasma extracellular vesicles enriched for neuronal origin. Aging Cell.

[230] Gocmez SS, Gacar N, Utkan T, Gacar G, Scarpace PJ, Tumer N (2016). Protective effects of resveratrol on aging-induced cognitive impairment in rats. Neurobiol Learn Mem.

[231] Cicero AFG, Ruscica M, Banach M (2019). Resveratrol and cognitive decline: a clinician perspective. Arch Med Sci.

[232] Harrison DE, Strong R, Sharp ZD, Nelson JF, Astle CM, Flurkey K, Nadon NL, Wilkinson JE, Frenkel K, Carter CS, Pahor M, Javors MA, Fernandez E, Miller RA (2009). Rapamycin fed late in life extends lifespan in genetically heterogeneous mice. Nature.

[233] Majumder S, Caccamo A, Medina DX, Benavides AD, Javors MA, Kraig E, Strong R, Richardson A, Oddo S (2012). Lifelong rapamycin administration ameliorates age-dependent cognitive deficits by reducing IL-1beta and enhancing NMDA signaling. Aging Cell.

[234] Martin-Montalvo A, Mercken EM, Mitchell SJ, Palacios HH, Mote PL, Scheibye-Knudsen M, Gomes AP, Ward TM, Minor RK, Blouin MJ, Schwab M, Pollak M, Zhang Y, Yu Y, Becker KG, Bohr VA, Ingram DK, Sinclair DA, Wolf NS, Spindler SR, Bernier M, de Cabo R (2013). Metformin improves healthspan and lifespan in mice. Nat Commun.

[235] Teng Z, Feng J, Qi Q, Dong Y, Xiao Y, Xie X, Meng N, Chen H, Zhang W, Lv P (2021). Long-term use of metformin is associated with reduced risk of cognitive impairment with alleviation of cerebral small vessel disease burden in patients with type 2 diabetes. Front Aging Neurosci.

[236] Kodali M, Attaluri S, Madhu LN, Shuai B, Upadhya R, Gonzalez JJ, Rao X, Shetty AK (2021). Metformin treatment in late middle age improves cognitive function with alleviation of microglial activation and enhancement of autophagy in the hippocampus. Aging Cell.

